# The absence of SigX results in impaired carbon metabolism and membrane fluidity in *Pseudomonas aeruginosa*

**DOI:** 10.1038/s41598-018-35503-3

**Published:** 2018-11-21

**Authors:** Maud Fléchard, Rachel Duchesne, Ali Tahrioui, Emeline Bouffartigues, Ségolène Depayras, Julie Hardouin, Coralie Lagy, Olivier Maillot, Damien Tortuel, Cecil Onyedikachi Azuama, Thomas Clamens, Cécile Duclairoir-Poc, Manuella Catel-Ferreira, Gwendoline Gicquel, Marc G. J. Feuilloley, Olivier Lesouhaitier, Hermann J. Heipieper, Marie-Christine Groleau, Éric Déziel, Pierre Cornelis, Sylvie Chevalier

**Affiliations:** 1 Normandie Université, Université de Rouen Normandie, Laboratoire de Microbiologie Signaux et Micro-environnement, LMSM EA 4312 Evreux, France; 2 Normandie Université, Université de Rouen Normandie, Laboratoire Polymères Biopolymères Surfaces, PBS, UMR, 6270 CNRS Mont-Saint-Aignan, France; 3 Department of Environmental Biotechnology, UFZ Helmholtz Centre for Environmental Research, Leipzig, Germany; 4 INRS-Institut Armand-Frappier, Laval, Québec, Canada

## Abstract

In *Pseudomonas aeruginosa*, SigX is an extra-cytoplasmic function σ factor that belongs to the cell wall stress response network. In previous studies, we made the puzzling observation that *sigX* mutant growth was severely affected in rich lysogeny broth (LB) but not in minimal medium. Here, through comparative transcriptomic and proteomic analysis, we show that the absence of SigX results in dysregulation of genes, whose products are mainly involved in transport, carbon and energy metabolisms. Production of most of these genes is controlled by carbon catabolite repression (CCR), a key regulatory system than ensures preferential carbon source uptake and utilization, substrate prioritization and metabolism. The strong CCR response elicited in LB was lowered in a *sigX* mutant, suggesting altered nutrient uptake. Since the absence of SigX affects membrane composition and fluidity, we suspected membrane changes to cause such phenotype. The detergent polysorbate 80 (PS80) can moderately destabilize the envelope resulting in non-specific increased nutrient intake. Remarkably, growth, membrane fluidity and expression of dysregulated genes in the *sigX* mutant strain were restored in LB supplemented with PS80. Altogether, these data suggest that SigX is indirectly involved in CCR regulation, possibly *via* its effects on membrane integrity and fluidity.

## Introduction

*Pseudomonas aeruginosa* is an opportunistic pathogen causing infections in immune-compromised persons and the main etiological agent of chronic infections strongly compromising the health of cystic fibrosis individuals^1^. The 6.3 mb genome of *P. aeruginosa* PAO1 encoding 5,570 predicted open reading frames (ORFs), contains a strikingly high proportion of two-component regulatory systems (2.1%) and transcriptional regulators (7.2%)^2^. These regulators have been hypothesized to provide the regulatory framework for the remarkable environmental adaptability of *P. aeruginosa*. Together with two-component (TCS) and quorum sensing (QS) systems, alternative sigma factors are among the most important regulators of *Pseudomonas*. Sigma factors are multi-domain subunits of bacterial RNA polymerase (RNAP) that play critical roles in transcription initiation, including the recognition and opening of promoters as well as the initial steps in RNA synthesis. While the primary sigma factor σ^70^ ensures the bulk of transcription during growth, a major strategy used by bacteria to differentially regulate gene expression consists in modifying the RNAP promoter specificity by means of alternative sigma factors. Among these factors, extra-cytoplasmic function sigma factors (ECFσ) constitute the most abundant group identified based on their homology with the primary sigma factor σ70^3^. Upon environmental stresses or specific inducing factors, signalling networks result in the release of the sigma factors, which then recruit the RNAP catalytic core and trigger gene expression of dedicated target regulons reprogramming, and triggering adaptive responses for the survival of the cell. *P. aeruginosa* PAO1 has 19 ECFσ factors^4^, including SigX that was recently described as a new cell wall stress response ECFσ^5,6^. SigX is a master regulator of bacterial adaptation that impacts more than 250 genes, including genes involved in adaptation/protection, heat shock response, chemotaxis, motility/attachment, virulence and virulence-associated genes linked to the protein secretion/export apparatus or secreted factors^7–10^. Importantly, SigX is involved in modulation of fatty acid (FA) and phospholipid metabolisms in *P. aeruginosa* PA14^9–11^, and overexpression of SigX results in “swollen” cells that contain higher amounts of shorter chain FA with more fluid membrane^11^. Interestingly, SigX shares 49% similarity with SigW, a membrane-fluidity homeostasis regulator of the Gram-positive *Bacillus subtilis* induced by factors that affect cell wall biosynthesis^12^.

In previous studies, we made the puzzling observation that the growth of *P. aeruginosa sigX* mutant was severely affected in lysogeny broth (LB), but not in M9-glucose minimal medium (M9G)^8,13^. Whereas M9G contains glucose as sole carbon source, the rich undefined LB medium is a complex mixture of substances, among which the most abundant carbon sources are small peptides and amino acids brought by tryptic digest of casein and yeast extract, as well as dicarboxylic acids such as succinate^14^. Since SigX is involved in membrane homeostasis by regulating its fluidity, possibly impacting nutriments uptake, we raised the hypothesis that growth alterations of the *sigX* mutant observed in LB could be linked to changes in some important metabolic pathways due to membrane alterations.

## Results and Discussion

### The absence of SigX results in large shifts in genes expression and proteins synthesis

Transcriptomic and proteomic analyses based on comparison between *P. aeruginosa* H103 and PAOSX (*sigX* mutant) grown in LB were performed. Global transcriptome analysis revealed a total of 758 expressed genes (*p* < 0.05 by Empirical Bayes statistical test) that differed by more than 2-fold (Supplementary Table S1), indicating that the deletion of *sigX* exerts a large and global impact on gene expression during growth in LB. Of these genes, 338 were down-regulated in the mutant while 420 were up-regulated, corresponding to nearly 14% of the total PAO1 genome. Sixteen genes that were differentially expressed between the WT H103 and PAOSX were further selected for validation using qRT-PCR, showing good correlation with microarrays data with a squared Pearson’s correlation coefficient of 0.83 (Supplementary Fig. S1). These results are in line with the previous study made on PAOSX grown in M9G^8^, and others that were conducted with a PA14 *sigX* mutant and overproducing strains^9^. Indeed, the transcriptome data in this study (PAO1 *sigX* mutant grown in LB medium containing 171 mM NaCl) overlapped with 26.4% and 15.7% of the genes identified in PA14 *sigX* mutant and SigX overexpressing strain grown in LB containing 8 mM NaCl, respectively^9^ (Supplementary Figure S2A). The transcriptomic data set of the *sigX* mutant grown in M9G^8^ overlapped with 17.7% of the genes identified in this study. A total of 15 common genes were identified in all four transcriptomes (Supplementary Figure S2A). The results of this transcriptional analysis contrast with the fact that most ECFσ exhibit a relatively small regulon^10^, with the exception of AlgU in *P. aeruginosa*, the homolog of *E. coli* RpoE^15^. It is worth to mention that, in addition to SigX, AlgU is also an ECFσ that is involved in the envelope (periplasmic) stress response in *P. aeruginosa*^4,10^. However, a large proportion of the differences observed in gene expression may probably be due to indirect effects caused by the absence of *sigX*, as suggested by the large number of genes up-regulated in PAOSX mutant.

The global proteome analysis led to identify 1055 +/− 49 and 1075 +/− 23 proteins in case of H103 and PAOSX, respectively, corresponding to about 19% of *P. aeruginosa* total proteome, out of which 92 proteins were displaying a significant change in abundance between the two strains (Supplementary Table S2). About 2/3 of these proteins, *i.e*. 54 out of 92 proteins, are encoded by genes that were also differentially expressed in transcriptomic data, and depicted as a VENN diagram (Supplementary Figure S2B). Such discrepancy between protein and transcript abundances can be at least partly explained by the technics used, since membrane proteins in particular, are quite difficult to extract. However, based on a hyper-geometrical testing, the overlap between the proteomic and transcriptomic data was shown to be significant (*p*-value = 7.5 × 10^−23^). Furthermore, we analyzed the overlap of our proteome data with the proteome of PA14 SigX overexpressing strain^11^. Only 8 proteins were present in the two proteomes (Supplementary Figure S2C), a result that can be at least partly explained by the different approaches used (2-DE or LTQ-orbitrap), the growth medium (LB containing 171 or 85 mM NaCl) or the *P. aeruginosa* strain (PAO1 or PA14).

**Figure 1:**
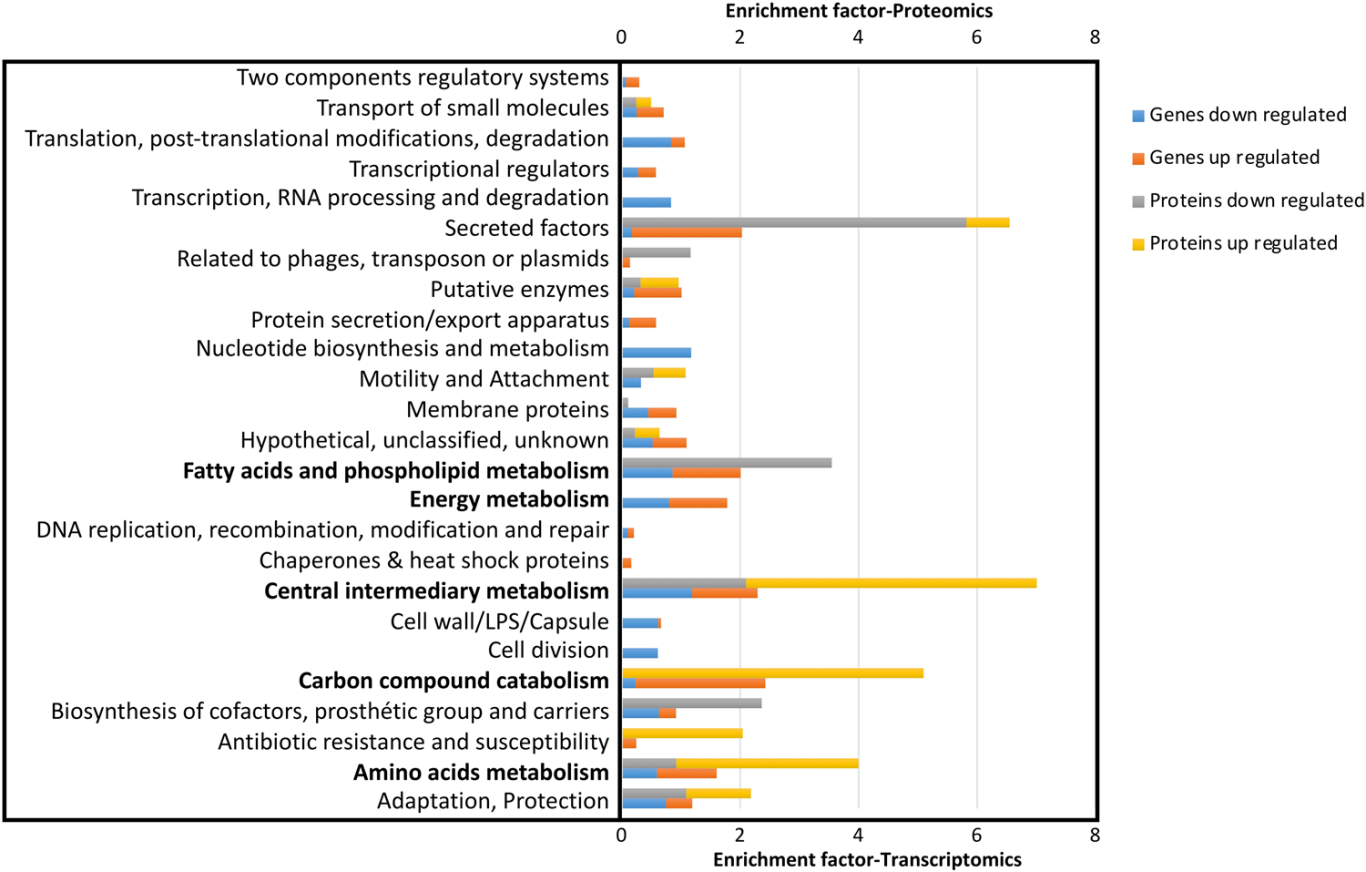
Functional classification of genes and proteins that are over- and under-represented in *P. aeruginosa* PAOSX compared to H103. The PseudoCAP annotation (www.pseudomonas.com)^16^ was used to categorize the members of the significantly differentially abundant genes or proteins, and enrichment of specific classes of genes or proteins relative to their distribution in PAO1 is displayed as the enrichment factor. The functional classes that will be discussed are indicated in bold.

Based on a PseudoCAP analysis^16^, the most striking result is the dysregulated expression of numerous proteins and genes involved in several functional classes related to transport and metabolism (energy, carbon compound, amino acids, intermediary, FA and phospholipids (Fig. 1), suggesting global and drastic changes in the physiology and nutritional metabolism of the *sigX* mutant. Since these alterations in gene and protein expression could at least partly explain the growth defects of PAOSX in LB, we focused subsequent analysis on selected genes from these major categories (Supplementary Table S3, Fig. 1).

### Transporters of small molecules and membrane proteins

The expression of 52 membrane transporters or transport-associated proteins was affected in the *sigX* mutant compared to H103 (Supplementary Table S3). For example, the expression of genes encoding the specific porins OprB, OprE, OprH, OpdC^17^, as well as the copper import TonB-dependent transporter OprC^18^, was reduced by 2 to 7.7-fold (Supplementary Table S3). Similar data were previously reported when PAOSX was grown in M9G^8,13^. Conversely, genes encoding OpdQ (a putative nitrate transporter^19^) and OpdP (also called OpdD, a glycine-glutamate dipeptide transporter) were increased by 14.8 and 3.2-fold, respectively in the *sigX* mutant strain compared to H103 WT. Expression of many carboxylic acids, carbohydrates and amino acids inner membrane (IM) transporters was also dysregulated. For example, the genes encoding the malonate transporter MadL/MadM and the dicarboxylic acid transporter PcaT were strongly up-regulated in the *sigX* mutant. This was noticeably also the case of genes, whose products are involved in the metabolization of these substrates (PA0208-PA0214 and PA0226-PA0235, respectively) (Supplementary Table S3, see section carbon metabolism). By contrast, PA1183 and PA4616 encoding the major DctA C_4_-dicarboxylate transporter and a putative C_4_-dicarboxylate binding protein of the DctP family, respectively, were down regulated in the *sigX* mutant (Supplementary Table S3). Similarly, the expression of all the genes in the *gltBFGK-oprB* operon, which are involved in glucose uptake^17^, is decreased in PAOSX, suggesting a reduced glucose supply in PAOSX. The operons PA1946-PA1948 (*rbsBAC*) and PA2338-PA2341 (*mltEFGK*), encoding components of a ribose transporter and mannose/mannitol transporters, respectively, were up-regulated in PAOSX. The *putA* and *putP* genes involved in the utilization of proline are also down-regulated in the PAOSX strain. The expression of several genes coding for components of ABC transporters, many of them are selective for amino acids, was increased in the *sigX* mutant, including *agtAB*, *aatPJ*, *braCG* (for branched-chain amino acids uptake), PA5094-PA5099 (for histidine uptake), and *opuC* coding for a glycine betaine transporter involved in osmoprotection (Supplementary Table S3). Taken together, expression of many genes coding for OM porins as well as IM transporters was dysregulated in the *sigX* mutant strain, suggesting that PAOSX could be affected in nutrients import in LB.

### Fatty acid and phospholipid metabolisms

Several genes involved in FA biosynthesis showed a markedly decreased expression in the *sigX* mutant (Supplementary Table S3; Supplementary Figure S3). Several steps of the initiation and elongation of FA biosynthesis are impacted since the expression of *fabD*, *fabY*, *fabB*, *fabA* and *fabZ* genes was clearly reduced in the *sigX* mutant. These data are in line with previous studies performed on a PA14 *sigX* mutant^9,10^ or a SigX overproducing strain^11^, where ChIP-sequencing assays^9,10^ or proteomic data^11^, respectively, led to the conclusion that at least some of these genes could be direct targets of SigX. Proteome analysis further indicated that the expression of FabG, FabD and FabY was also decreased at protein level (Supplementary Table S3). In addition, the expression of AccA, that catalyzes, together with AccD, the first step in FA biosynthesis, is also affected (Supplementary Table S3). The reaction catalyzed by AccAD requires the presence of biotin carboxyl carrier protein that is produced by the carboxylation of the product of *accB* (PA4847) by a biotin carboxylase encoded by *accC* (PA4848). Another putative biotin carboxylase homolog sharing 55% identity with AccC is encoded by PA0494, a gene lying in the PA0493-PA0496 operon, whose transcript level is also strongly decreased (>6×) in PAOSX (Supplementary Table S3). Moreover, the product of PA0493 shares 40% identity with AccB. Although the precise function of the proteins encoded by PA0493 and PA0494 is unknown, these data further suggest that the first step in FA biosynthesis is somehow affected by the *sigX* mutation.

### Carbon metabolism

**Figure 2:**
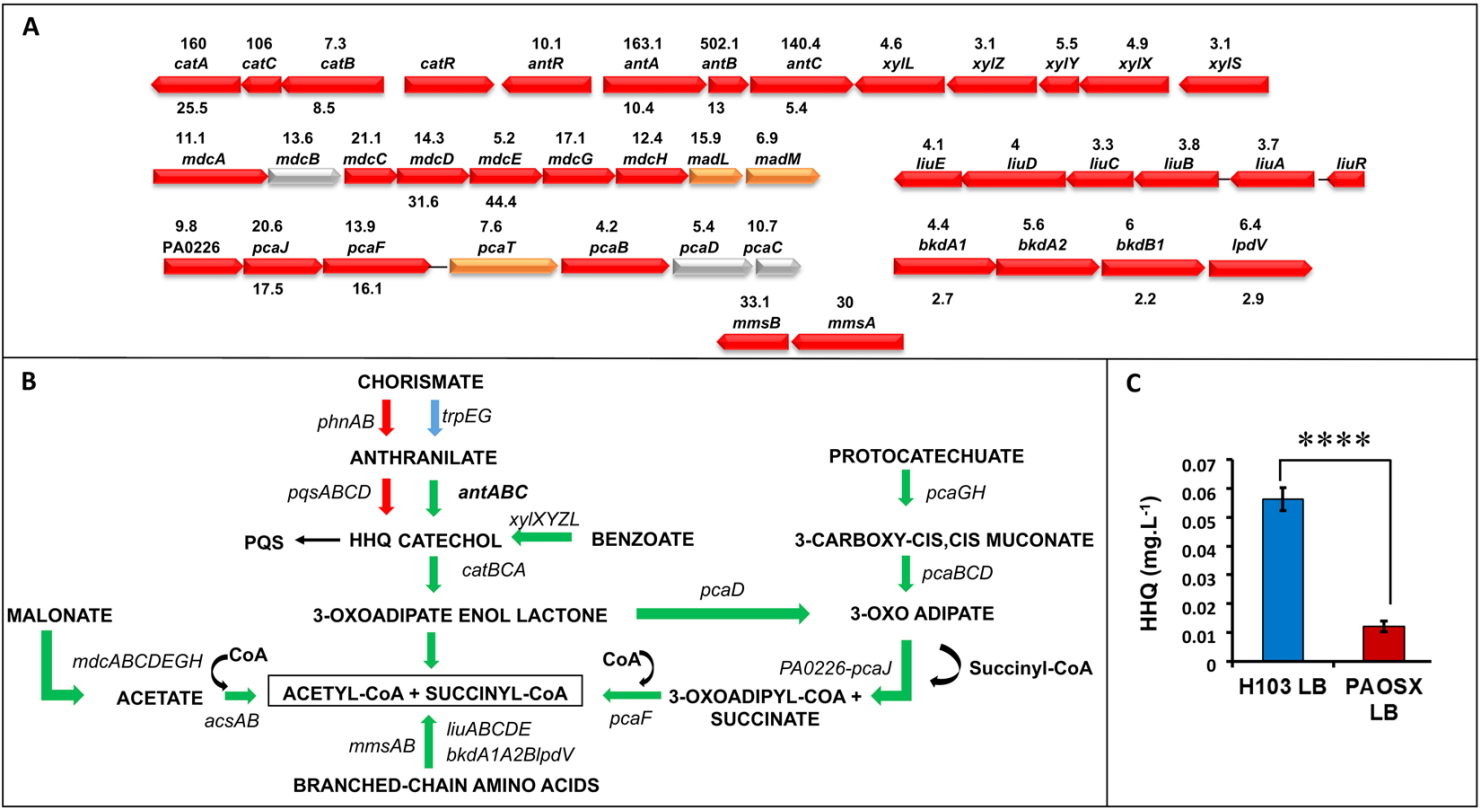
The carbon and anthranilate metabolic pathways are affected by the *sigX* deletion. Major carbon catabolic pathways leading to acetyl-CoA production are depicted. (**A**) Operons that are dysregulated in the *sigX* mutant strain are depicted as indicated on pseudomonas.com^16^. The fold change between PAOSX and H103 gene and protein expression is indicated above and below the genes, respectively. Colours correspond to protein localization: red: cytoplasmic; orange: cytoplasmic membrane; yellow: periplasmic; grey: unknown). (**B**) Major metabolic pathways that are dysregulated in PAOSX compared to H103. Arrows in green: up-regulated pathways; in red, down-regulated pathways, in blue: unaffected pathways. (**C**) HHQ production quantification (mg.L^−1^) by LC/MS in PAOSX and H103. Statistics were achieved by unpaired t-test: ****p < 0.0001.

One of the most striking data of our global studies is the strong up-regulation of the *ant, cat*, *pca* and *xyl* operons that are involved in carbon metabolic pathways (Supplementary Table S3, Fig. 2A). In *Pseudomonas* spp. the degradation of the aromatic amino acid anthranilate is achieved by the anthranilate dioxygenase complex (*antABC* operon), which is involved in the conversion of anthranilate to catechol (Fig. 2B)^20^. Catechol is further metabolized by the *cat* and *pca* gene products to generate intermediates of the tricarboxylic acid cycle (TCA) including acetyl-CoA^21^. Catechol is also a product of the benzoate degradation pathway (*xyl* operon). The *antABC* genes are positively regulated by the LysR-type regulator AntR^20^, whose gene expression is also 10-fold up-regulated in the *sigX* mutant. In parallel, the expression of the *pqs* operon (for *Pseudomonas* quinolone signal), that is involved in a quorum-sensing system allowing cell-to-cell communication, was reduced at both mRNA and protein levels. Taken together, these results suggest that anthranilate is preferably channelled to feed the TCA cycle rather than being used for biosynthesis of HHQ (2-heptyl-4-hydroxyquinoline), the precursor of the quorum sensing PQS pathway^22^, or tryptophan. This was further confirmed by HHQ quantification using LC-MS/MS, since this compound was about 5-fold lower accumulated in PAOSX compared to the WT strain (Fig. 2C). It is noteworthy here, that while PQS levels were still very low (near limit of detection) at this early time point, the mutant was also producing less than the WT strain. In addition to the redirection of anthranilate metabolism, other genes involved in metabolic pathways leading to the production of acetyl-CoA were strongly up-regulated in the *sigX* mutant. This was the case for the *mdcABCDEGH* and *acsAB* operons, whose products catalyse the conversion of malonate or acetate, respectively into acetyl-CoA (Fig. 2B).

### Amino acid metabolism

Most genes belonging to the amino acids metabolism functional class are up-regulated in the *sigX* mutant, suggesting an indirect effect of *sigX* deletion on the expression of these genes (Supplementary Table S3). Strikingly, many operons encoding enzymes belonging to the degradation pathway of the branched-chain amino acids leucine, valine and isoleucine were strongly up-regulated. This is particularly exemplified in the case of L-leucine conversion to acetyl-CoA (Supplementary Table S3, Fig. 2A,B)^23^. The Ldh leucine dehydrogenase converts leucine in 4-methyl-2-oxo-pentanoate, which is further converted to isovaleryl-CoA by the combined action of BkdA1 and BkdA2, BkdB, and LpdV. The different Liu proteins are involved in the further catabolism of isovaleryl-CoA leading to the final products acetate and acetyl-CoA^23^. Noticeably, some of the corresponding proteins were also more abundant in the *sigX* mutant (Supplementary Table S3). Furthermore, the *hutUH* and *hutIG* operons, which encode enzymes involved in the degradation of histidine into glutamate^24^ showed an increased expression in the *sigX* mutant (Supplementary Table S3). The most up-regulated gene is *hutH* (more than 30-fold) that codes for the histidine ammonia lyase, which performs the first step of histidine catabolism, generating urocanate and ammonium^24^. The genes PA5095-5096-*hutT* and PA5099 coding for associated transporters are likewise up-regulated in PAOSX. This global metabolic reorientation leading to the induction of the TCA cycle may be a compensatory mechanism triggered by PAOSX to cope with energy depletion.

### Energy generation

**Figure 3:**
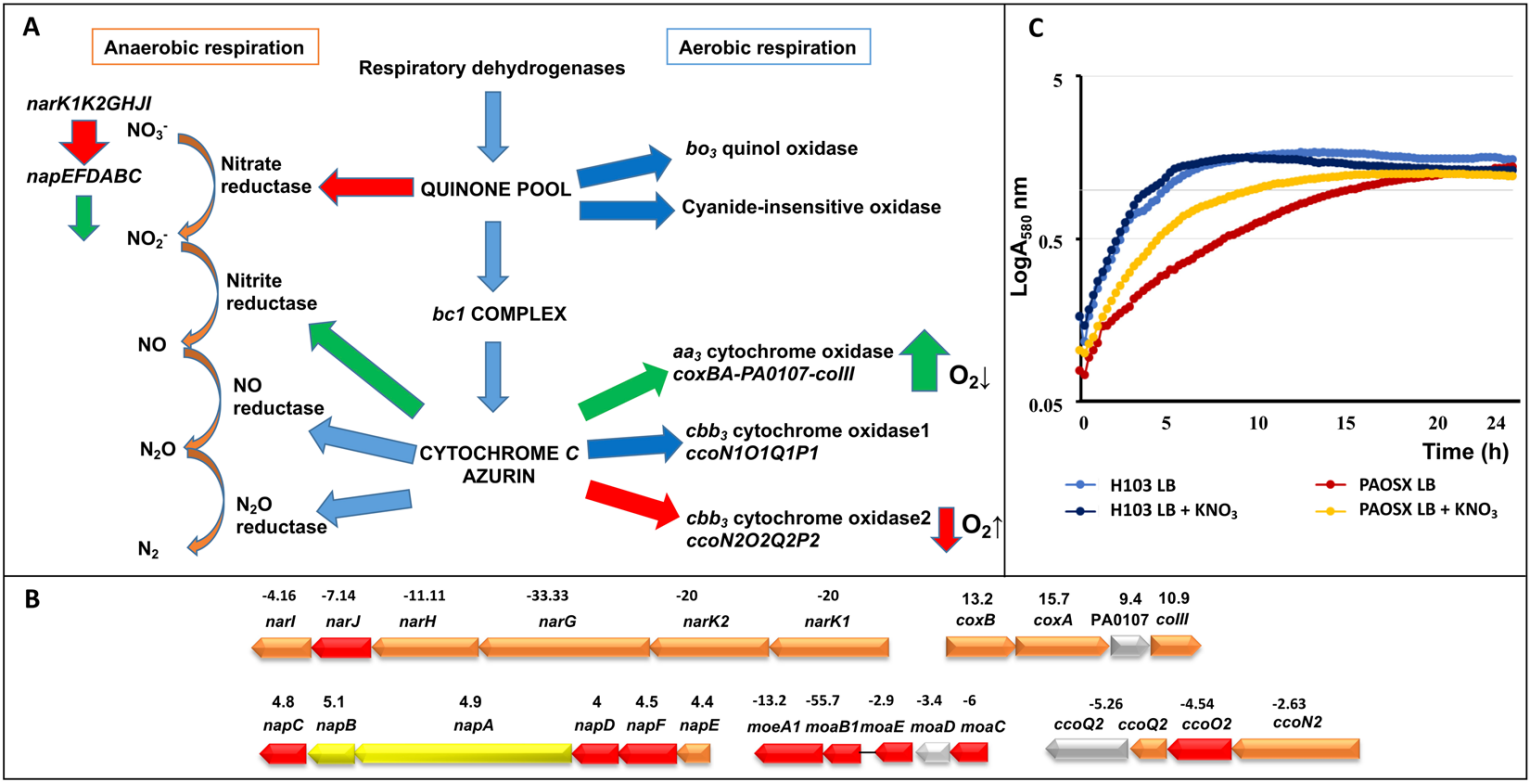
The respiratory network is affected in the *sigX* mutant. (**A**) Major aerobic and anaerobic pathways. Arrows in green: up-regulated pathways in PAOSX; in red, down-regulated pathways, in blue: unaffected pathways. (**B**) Operons that are dysregulated in the *sigX* mutant strain (fold change is indicated above the genes) are depicted as indicated on pseudomonas.com^16^. Colours correspond to protein localization: red: cytoplasmic; orange: cytoplasmic membrane; Yellow: periplasmic; grey: unknown). (**C**) Growth of H103 and PAOSX in LB or LB supplemented with KNO_3_.

Figure 3A presents the different respiratory electron transport chains of *P. aeruginosa*^25^ and the changes observed in PAOSX when grown in LB. The *aa3* genes encoding the low O_2_ affinity cytochrome oxidase were up-regulated in PAOSX while one set of the *cbb3* genes coding for an oxidase with high-affinity for O_2_ were down-regulated (Fig. 3A). Interestingly, the *aa3*-type cytochrome c oxidase was shown to be induced in response to carbon starvation^25^ and facilitates survival in nutrient starvation conditions^26^, suggesting that PAOSX may encounter a nutrient stress. In addition, the expression of the gene encoding the aerotaxis sensor Aer that is positively regulated by the anaerobic transcriptional regulator Anr, was lowered in the *sigX* mutant. The genome of *P. aeruginosa* contains two *cbb3* oxidase operons, *ccoN1O1Q1P1* and *ccoN2O2Q2P2*. While the first one is always expressed, the *ccoN2O2Q2* operon whose expression is reduced in PAOSX, is induced by stress, nutrient limitation and low oxygen conditions^25^ (Supplementary Table S3, Fig. 3B). Furthermore, the *nar* genes, encoding the membrane respiratory nitrate reductase that generates a H^+^ gradient, are strongly down-regulated in PAOSX, while the *nap* genes, which code for the periplasmic nitrate reductase that does not contribute to the building of a proton motive force, are up-regulated in the *sigX* mutant. Consistently, the genes encoding the NarXL TCS were down-regulated in PAOSX. Indeed, under anaerobic growth conditions, NarL was shown to directly repress expression of the periplasmic nitrate reductase while inducing maximal expression of the membrane nitrate reductase^27^. Under static conditions, the growth defect of PAOSX in LB was partially restored by supplementation with KNO_3_ (Fig. 3C). It is known that nitrate induces the *nar* operon under anaerobic conditions via the Anr and Dnr regulators and the NarXL TCS^28^. This suggests that the addition of nitrate under our low aeration condition may stimulate the growth of the *sigX* mutant by increasing the transcription of the *narK1K2GHJI* operon. Furthermore, we observed that the transcript level of the PA3914-PA3918 operon (*moeA1-moeB1-moaE-moaC-moaD*) was strongly down-regulated in the PAOSX mutant. This gene cluster is involved in the biosynthesis of the molybdenum cofactor, which is needed for the activity of all nitrate reductases^29^. This decreased expression was also linked to a lower expression of the *modA* gene involved in molybdenum acquisition^30^. These alterations suggest that decreased Nar-dependent generation of ATP might contribute to the mutant growth defect at least in anaerobic but probably not in aerobic conditions, in which the transcriptomic and proteomic analysis were carried out. In the latter condition, the dysregulation of the O_2_-dependent oxidases may suggest that O_2_ perception or diffusion may be different between the two strains.

### The absence of SigX results in alterations in the Crc, Hfq and CbrAB regulatory networks

In numerous bacterial species, the uptake and utilization of carbon compounds is controlled in a hierarchical manner by carbon catabolite repression (CCR). Broadly, CCR prevents the utilization of less-preferred carbon sources for as long as the preferred one is not fully consumed. In Pseudomonads the presence of organic acids such as succinate triggers CCR response, which leads to repression of catabolic genes required for the consumption of other carbon sources. CCR is thought to be elicited mainly through a regulatory system based on Crc and Hfq together with CrcZ, a small RNA that belongs to the regulon of the CbrAB TCS and which is reported to antagonize the effects of both regulatory proteins^31–33^. When CCR is not needed, CrcZ forms a complex with Hfq and possibly also with Crc, which renders these proteins unavailable to repress translation initiation of their targets^31,33,34^. The strong repression exerted by the Crc/Hfq system during exponential growth in LB ceases when the cells reach the stationary phase. Since LB elicits a strong CCR response^31^, the involvement of these regulatory networks in the *sigX* mutant strain was therefore further investigated.

### Reduced expression and activity of the carbon catabolic regulator Crc in the sigX mutant strain

In *P. aeruginosa*, CCR is at least partly regulated by the Crc regulator^32,35,36^ Transcriptomic analysis performed during growth in LB revealed that *crc* (PA5332) was lower expressed in PAOSX compared to H103 (PAOSX/H103 = 0.31 +/− 0.06), a result that was confirmed by qRT-PCR (Supplementary Table S3). Interestingly, the gene expression profile of PAOSX exhibits several similarities with that of a *crc* mutant of *P. aeruginosa* during growth in LB^36^. Inactivation of *crc* modifies the expression of at least 57 genes in *P. aeruginosa* among which many are involved in the transport and assimilation of amino acids and sugars, or in energy metabolism^36^. Under our conditions, 48 of these Crc-regulated genes were significantly affected in the *sigX* mutant strain. Most of these genes encode proteins involved in the transport of glutamate or derivatives (porins OpdP, OpdQ) and aspartate (*aat* genes), branched-chain (*braC*) and aromatic (*aroP2*) amino acids, and organic acids (OpdH porin) (Supplementary Table S3). The stronger expression of these genes encoding components of transport systems is paralleled by that of genes involved in the assimilation of the amino acids valine, leucine and isoleucine (*bkdA1A2B, lpdV, liuABCD*), or phenylalanine and tyrosine (*phhAB* and *hpd*). Furthermore, Crc was previously shown to stimulate the expression of the low affinity C_4_-dicarboxylate transporter (DctA) that is involved in the uptake of succinate and fumarate, two of the preferential carbon sources used by *P. aeruginosa*^37^. Interestingly, *dctA* (PA1183) was strongly down-regulated in PAOSX, in agreement with the lower expression of *crc*. Finally, Crc mediates catabolite repression of *amiE*, which encodes a short-chain aliphatic amidase and can be used as a reporter gene for CCR^37^. Taken together, and since *amiE* expression was strongly increased at both the mRNA and protein levels in PAOSX (Supplementary Table S3), our data support that Crc-based regulation is strongly reduced in the *sigX* mutant compared to H103 when grown in LB. Crc regulates gene expression post-transcriptionally^32,35,36^ However, much of what is known about CCR comes from work on mutants lacking the Crc protein in *P. aeruginosa*^32,36^. Due to the technics used herein (microarrays and proteomics), some Crc targets, the expression of which was disturbed, were not identified at the protein level to observe post-transcriptional regulation. However, initially deemed to act as a translational regulator, Crc is devoid of any RNA binding activity in the absence of the regulator Hfq^38^, indicating that Crc and Hfq cooperate in catabolite repression. Very recently, Crc was shown to enhance the stability of Hfq/Crc/RNA complexes, which can explain its facilitating role in Hfq-mediated translational repression, and to prioritize the function of Hfq toward utilization of favored carbon sources^39^. Thus, in lowering the transcripts of *crc* in PAOSX, and possibly also Crc abundance (even if not shown in the proteomic data), it is possible that Hfq may not be directed on these targets mRNA, leading to alleviate its repression on its CCR targets in the *sigX* mutant.

### Hfq repressor activity is lowered in the sigX mutant strain

In *P. aeruginosa* PAO1, the RNA chaperone Hfq was identified as the main post-transcriptional regulator of CCR^31^. A transcriptomic analysis of a *hfq* mutant strain revealed that 15% of the genes were differentially regulated^40^. Many of them encode proteins involved in carbon compound catabolism and were up-regulated in the absence of Hfq^40^. In a very recent study, Kambara *et al*. found that (i) Hfq associates with hundreds of nascent transcripts in *P. aeruginosa*, (ii) Hfq and Crc frequently act together co-transcriptionally and (iii) association of Crc with nascent transcripts is Hfq-dependent^41^. Interestingly, 102 genes whose expression was dysregulated in a *hfq* mutant^31,40^, were similarly affected in PAOSX, at the transcript level, and for 22 of them, at the protein level (Supplementary Table S3, Hfq^(1)^ and Hfq^(2)^). Among them, the Crc/Hfq regulated genes, which are mostly involved in amino acids metabolism, showed an increased expression in the *sigX* mutant. Fifty-three genes involved in carbon metabolism that were over-expressed in the *hfq* mutants^31,40^ were also strongly up-regulated in PAOSX (Supplementary Table S3). This was particularly significant in the case of the *ant, cat*, and *xyl* operons, and of *amiE*. Five additional Hfq targets (*opdO, mdcD, mdcE, pagL, pycB*), previously identified by ChIP-seq^41^. were showing altered mRNA and/or protein abundances in PAOSX (Supplementary Table S3, Hfq^(3)^). As the transcript level of *hfq* was not modified in the mutant (PAOSX/H103 = 0.93 +/− 0.03), and as the proteomic data did not allow to conclude about Hfq protein amount (not detected in all replicates), our results suggest that Hfq protein amount or activity itself might be reduced. Since reduced activities of both Crc and Hfq might occur in PAOSX, this prompted us to investigate the activity of the CrcZ sncRNA, whose transcription is augmented when CCR is alleviated^31^. Interestingly, the level of CrcZ was significantly increased in PAOSX compared to the wild type (PAOSX/H103 = 2.39 +/− 0.78), suggesting reduced levels of free Hfq and Crc in the cytosol due to their sequestration by this sncRNA^42^.

### The CbrAB regulatory system is over-activated in the sigX mutant

A large number of genes transcriptionally down-regulated in a *cbrB* mutant of *P. aeruginosa* grown in LB broth^36^ were globally up-regulated in the *sigX* mutant strain (Supplementary Table S3, CbrAB), suggesting that the CbrAB pathway is over-activated in PAOSX. Apart from the *crcZ* promoter, known direct CbrB targets include the *lipA* and *hutU* promoters^43,44^. Accordingly, their transcripts abundance was increased in PAOSX. Furthermore, the *pha* genes, whose products allow bacteria to store excess carbon into the carbon-storage polyhydroxyalkanonates (PHA)-granules^45^, are up-regulated in the *sigX* mutant (Supplementary Table S3). PHA are produced by various bacterial species as intracellular carbon and energy storage materials under nutrient-limited conditions in the presence of excess carbon sources, and their synthesis is inhibited by the Crc/Hfq system during exponential growth^46^.

Taken together, our data suggest that strongly altered regulations of metabolic pathways lead to a reduced CCR in the *sigX* mutant compared to H103 WT strain. More precisely, the CbrA/B system was found to be over-activated in PAOSX, leading to a higher production of CrcZ which in turn would sequester the translational regulators Hfq and Crc during growth in LB. In addition, transcription of *crc* was found to be down-regulated in PAOSX, further stimulating the expression of the Crc regulon. During growth in M9G, CCR is strongly reduced since glucose is the sole carbon source available: this could explain why PAOSX exhibits no growth defect under these conditions^8,13^.

### The growth and metabolic networks dysregulation of the sigX mutant are restored by polysorbate 80 supplementation

**Figure 4:**
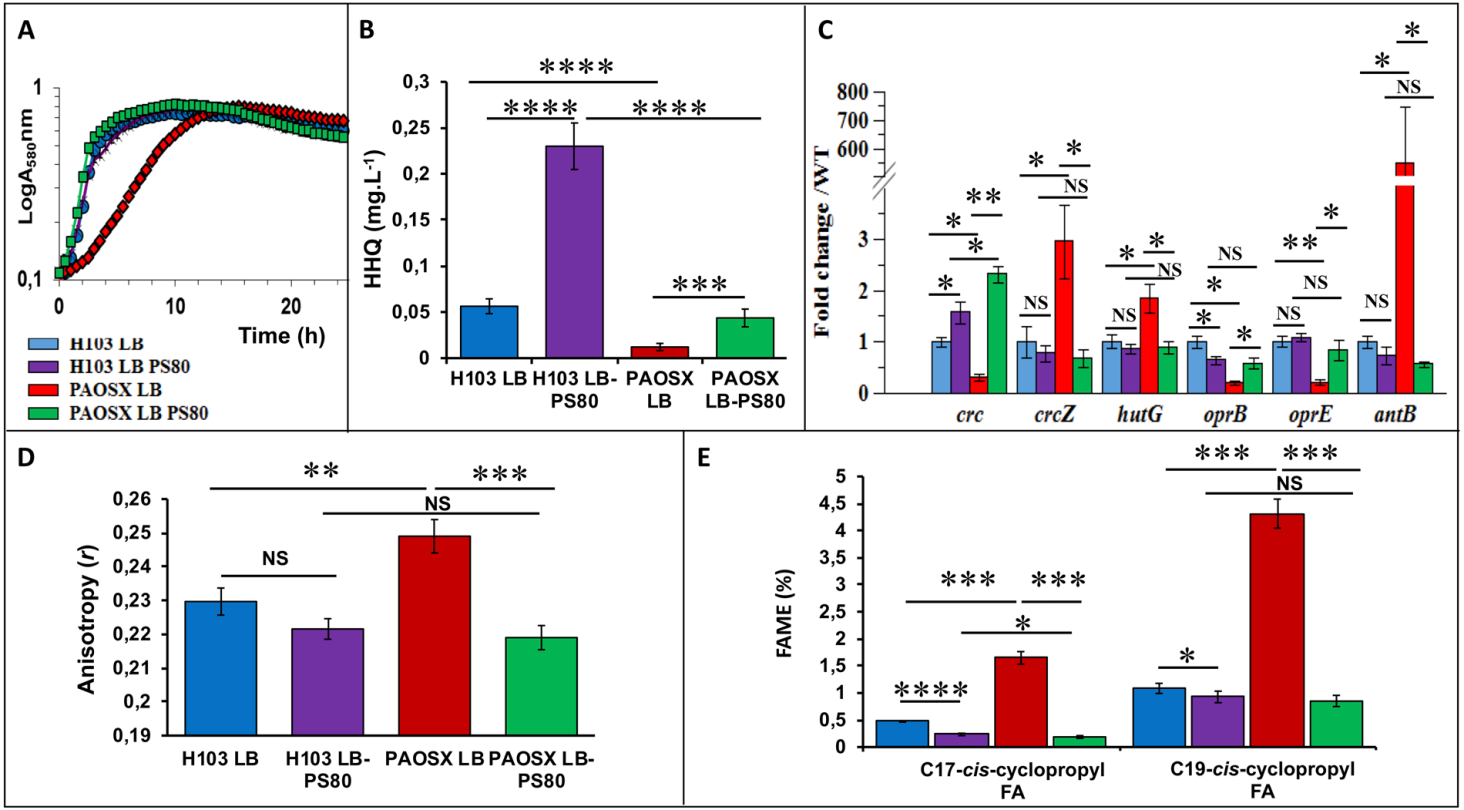
Polysorbate 80 (PS80) restores PAOSX growth, HHQ production, expression of Crc-Hfq target genes, and membrane alterations. (**A**) Growth kinetics of H103 (blue, purple) and PAOSX (red, green) grown in LB or LB-PS80, respectively. (**B**) Quantification of HHQ production by LC/MS of H103 (blue, purple) and PAOSX (red, green) grown in LB or LB-PS80, respectively. (**C**) qRT-PCR assayed on RNAs extracted from H103 (blue, purple) and PAOSX (red, green) grown in LB or LB-PS80, respectively. (**D**) Membrane fluidity (fluorescence anisotropy) of H103 (blue, purple) and PAOSX (red, green) grown in LB or LB-PS80, respectively. (**E**) Percentage of C_17_ and C_19_ cyclopropyl fatty acids (FAME analysis) extracted from H103 (blue, purple) and PAOSX (red, green) grown in LB or LB-PS80, respectively. Statistics were achieved by unpaired t-test: ****p < 0.0001; ***p = 0.0001 to 0.001; **p = 0.001 to 0.01; *p = 0.01 to 0.05; NS (Not significant), *p* ≥ 0.05.

As indicated above, the *sigX* mutant strain was strongly affected in the expression of nutrient transporters and in major metabolic regulatory pathways including CCR, suggesting that nutrient uptake would be disturbed. It is well known that detergents, at low concentration, can moderately destabilize the bacterial envelope resulting in a non-specific increased nutrient intake. Polysorbate 80 (PS80) can thus be used to allow microorganisms to uptake some nutrients they are unable to transport, as in the case of glucose in the probiotic bacterium *Bifidobacterium animalis* subsp. *lactis*^47^. The growth of PAOSX in LB was strongly affected compared to that of H103, but this growth defect was almost totally supressed by the addition of PS80 to LB (LB-PS80) (Fig. 4A). Similarly, the HHQ production decrease observed in PAOSX was restored to near WT level when the mutant strain was grown in LB-PS80 (Fig. 4B). We further investigated the effect of PS80 on the activity of Crc/Hfq/CbrAB networks by qRT-PCR on some selected target genes. When the *sigX* mutant was grown in LB-PS80, their expression level was restored at least partly to the WT level (Fig. 4C). Specifically, the expression of CrcZ was drastically and significantly reduced upon addition of the surfactant, suggesting that CbrA/B became less active. Consistently, the transcript levels of some members of the CbrAB, Crc and Hfq regulons, including *antB*, *hutG*, *oprE* or *oprB*, were also restored to WT levels, confirming the stimulation of CCR by PS80 in PAOSX. This was particularly spectacular in the case of *antB*, for which the decrease in the transcript level was dramatic. On the opposite, PS80 had no drastic effects on H103 growth (Fig. 4A) and Crc/Hfq/CbrAB-selected targets expression (Fig. 4C), excepted for HHQ, which amount was about 5-fold increased in presence of the detergent (Fig. 4B). These data raise the question of the mechanism by which PS80 acts on *P. aeruginosa*. Noticeably, it was previously shown that many Crc- and/or Hfq-repressed genes were expressed at a higher level when *P. putida* was grown in LB at 10 °C compared to 30 °C^48^. This was particularly the case for genes encoding proteins that are involved in transport and metabolism of some non-preferred carbon sources, suggesting that CCR may be less effective at low temperature. Interestingly, a *lptA* mutant (affected in phospholipid biosynthesis) displayed a lower membrane fluidity and a reduced PQS production^49^. Since cold-induced stress reduces membrane fluidity, and because a SigX overproducing strain displays an increased membrane fluidity^11^, we further investigated the effect of PS80 on the fatty acids composition of PAOSX membranes and its impact on membrane fluidity.

### Altered membrane lipid composition and fluidity may explain the dysregulation of metabolic networks in the sigX mutant

Membrane fluidity of H103 and PAOSX grown in LB and LB-PS80 was measured by anisotropy assays. Anisotropy of the *sigX* mutant was significantly higher than that of H103, confirming the lower membrane fluidity of PAOSX (Fig. 4D), in agreement with previous results, in which a SigX overproducing strain displayed a lower anisotropy compared to its corresponding WT strain, reflecting an increased membrane fluidity of this strain^11^. While no significant effect was observed in case of H103, (despite a slight increased membrane fluidity tendency), addition of 0.1% of PS80 restored the membrane fluidity of PAOSX to a level similar of that of the WT grown in LB or LB-PS80 (Fig. 4D). Interestingly, previous studies performed on plant cells showed that FAs externally added to the medium can be incorporated into phospholipidic membranes. PS80, which harbours in its hydrophobic part an esterified long-chain FA that is structurally related to C18Δ9 FA, can partially rescue the phenotype of an *Arabidopsis* mutant impaired in FA synthesis^50^. Since bacteria have the capacity to increase the fluidity of their membrane bilayer *via* the incorporation of more unsaturated FAs, or FA with analogous properties^51^, it is conceivable that addition of PS80 may restore, at least partly, the *sigX* mutant FAs content.

The main FAs identified in H103 WT strain were C18:1Δ11 *cis*-unsaturated fatty acid (UFA, 43%), C16:0 FA (38%) and C16:1Δ9 *cis*-UFA (12%), as previously reported^9,11^, while other FAs were poorly represented (below 3%) (Table 1). Compared to H103, the *sigX* mutant displayed lower percentage of C16:0 and C16:1Δ9 *cis*-UFA and a slight increase in C18:1Δ11 *cis* (Δ11) UFA, in line with a previous study showing that a *sigX*-overproducing PA14 strain displayed opposite percentages of these FA species^11^. However, as the degree of FA saturation was not strongly modified in PAOSX (0.73) compared to H103 (0.72), other factors may account for its lower membrane fluidity. Notably, the *sigX* mutant strain displayed also higher relative ratios of C17 and C19-*cis*-cyclopropyl containing FA (Table 1, Fig. 4E). These FAs decrease the mobility of reporter molecules within a membrane, and increase the tightness of packing within lipid bilayers, characteristics of a more rigid membrane^52^. The FA content of the *sigX* mutant was strongly modified when grown in LB-PS80. Thus, C18Δ9, the FA moiety of PS80 was over-represented in PAOSX exposed to PS80 (about 45% of the total FA content), suggesting a direct insertion of the molecule into its membranes. In addition, while the contents of C18:1Δ11 *cis* and C16:1Δ9 *cis* UFAs were strongly reduced (to about 12% and 1.8%, respectively), possibly a result of PS80 incorporation, the degree of FA saturation was globally lowered (0.57). Noticeably, PS80 modified also H103 membrane composition, leading to similar FA species amounts as in case of PAOSX grown in LB-PS80 (Table 1). Interestingly, PS80 addition reduced strongly the proportion of C17 and C19-*cis*-cyclopropyl containing FAs in PAOSX, and to lesser extend in H103 membranes (Fig. 4E). As a result, PAOSX and H103 grown in LB-PS80 displayed a similar membrane fluidity that can be related to similar FA composition and proportion. FA cyclopropanation is generally assumed to enhance the chemical and physical stability of membranes^53^. This adaptation was shown to occur in bacteria facing adverse environmental conditions^54^, such as tolerance to high osmotic pressure, high temperature, low pH, or nutrient deprivation. Altogether, these data suggest that addition of PS80 increased membrane fluidity in the *sigX* mutant, restoring at least some membrane functionalities that could be involved in CCR.

**Table 1:** Percentage of fatty acids species extracted from *P. aeruginosa* H103 and its isogenic *sigX* mutant strain PAOSX grown in LB or LB-PS80.

% FA	H103 LB	H103 LB-PS80	PAOSX LB	PAOSX LB-PS80
C16:0	38.42 +/− 0.15	32.23 +/− 1.58	36.41 +/− 0.35	32.68 +/− 0.65
C16:1Δ9-*trans*	1.32 +/− 0,51	2.07 +/− 0.36	1.37 +/− 0.32	2.7 +/− 1.0
C16:1Δ9-*cis*	12.39 +/− 0.23	2.62 +/− 0.07	7.51 +/− 0.14	1.83 +/− 0.07
C17-*cis*-cyclopropyl	0.47 +/− 0.013	0.23 +/− 0.022	1.65 +/− 0.11	0.19 +/− 0.02
C18:0	2.66 +/− 0.31	2.93 +/− 1.2	3.39 +/− 0.92	3.05 +/− 0.46
C18:1-*trans*	0.55 +/− 0.43	0	0	0
C18:1Δ9-*cis*	0	44.07 +/− 3.35	0	45.56 +/− 2.71
C18:1Δ11-*cis*	43.1 +/− 0.75	14.92 +/− 1.6	45.36 +/− 0.75	13.15 +/− 2.11
C19*-cis*-cyclopropyl	1.08 +/− 0.03	0.93 +/− 0.11	4.31 +/− 0.26	0.85 +/− 0.10

### Concluding remarks

**Figure 5:**
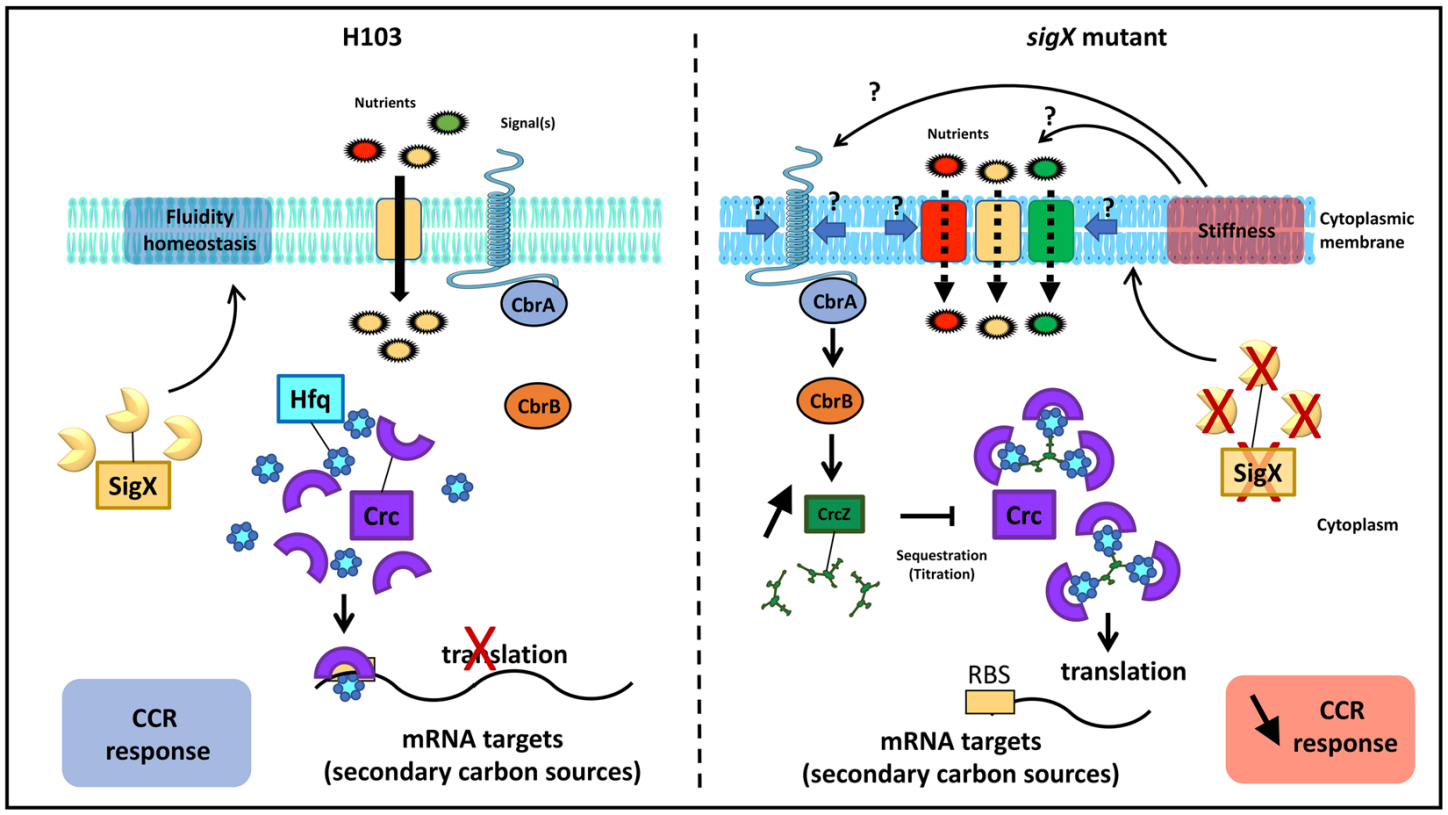
Membrane stiffness may account for lower CCR in the *sigX* mutant. In *P. aeruginosa* H103 grown in LB, in which the preferred carbon sources are present in non-limiting concentrations, CCR occurs through the Hfq/Crc/CrcZ pathway leading to repression of catabolite genes required for the consumption of other carbon sources^31,33,36^. The absence of SigX results in reduced membrane fluidity, a change that could disturb CbrA sensor activity, alter the anchorage and/or the activity of some membrane transporters and/or sensors involved in nutrient uptake. SigX seems thus to be indirectly involved in CCR regulation, possibly via its effects on membrane integrity and fluidity.

SigX is a multifaceted major ECF sigma factor of the RpoE-like family^4^ that has been proposed to maintain membrane functionality homeostasis^9,11,13^, in response to envelope stress signals^5–7^. This function is further supported by the fact that the Gram-positive *Bacillus subtilis* ECF sigma factor σW, which shares strong similarity with SigX, is a membrane-fluidity homeostasis regulator^55^. Similarly, the absence of SigX results in reduced membrane fluidity, a change that could alter the anchorage and/or the activity of some membrane transporters involved in nutrient uptake. Since CCR is only observed when the preferred carbon source is present at concentrations that do not limit growth, or at least when it is present in excess relative to other nutrients, it is possible that the reduced membrane fluidity of the *sigX* mutant may account, at least partly, for the reduced CCR that was observed in this strain during growth in LB, for instance by disturbing the activity of the CbrA membrane protein. Taken together, these data suggest that SigX is indirectly involved in CCR regulation, possibly via its effects on membrane integrity and fluidity (Fig. 5). How membrane fluidity can impact on the regulators that are involved in CCR (Crc/Hfq/CrcZ) is however not a trivial question. Further investigations are required to explore this hypothesis.

## Methods

### Bacterial strains and growth conditions

The strains used in this study were *P. aeruginosa* H103 (the prototrophic sequenced isolate strain PAO1)^56^ and its isogenic *sigX* deletion mutant PAOSX^7^. Bacteria were grown in LB containing 171 mM (10 g. L^−1^) NaCl on a rotary shaker (180 rpm) at 37 °C; growth was followed by A_580_ determination. When required, *P. aeruginosa* strains were grown in LB containing 0.1% polysorbate 80 (LB-PS80). For microaerobic growth, H103 and PAOSX were grown in LB containing 100 mM KNO_3_ during 24 h in static condition in microtiter plates (250 µl per well).

### Extraction of RNA, reverse transcription and quantitative real-time PCR (qRT-PCR)

Bacteria were grown in LB broth to mid-log phase (A_580_ = 0.4) before RNA extraction. Total RNA was prepared by the hot acid-phenol method as previously described^7^, with minor modifications. Briefly, cells were lysed, and RNA was extracted two times with an equal volume of acidic hot phenol and once with chloroform. RNA was ethanol precipitated, air dried, and dissolved in DEPC-treated water. Contaminating DNA was removed from total RNA by using 2 U of RNase-free TURBO DnaseI (InVitrogen) in a 50-μl mixture containing 6.25 mM MgCl_2_ and approximately 3 μg/μL^−1^ of total RNA. The reaction mixture was incubated 30 min at 37 °C, and DNase I was inactivated by adding 1 μL of 0.5 M EDTA for 10 min at 65 °C. The RNA concentration was determined spectrophotometrically at 260 nm. Quantitative reverse transcription-PCR (qRT-PCR) experiments were conducted as previously described^57^, using primers listed in Supplementary Table S4.

### Transcriptome analysis

The RNA concentration was determined by measuring the absorbance at 260 nm, and the quality of the RNA was assessed by spectrophotometry (NanoDrop ND-1000) and checked on a 1.2% agarose gel prior to use. Only samples showing A_260_/A_280_ and A_260_/A_230_ ratios above 2.0 were selected. RNA quality was then assessed with a Bioanalyzer 2100 (Agilent Technologies). Samples having an RNA Integrity Number (RIN) of 9 were retained. Ten μg of total RNA were used for each replicate with random hexamer primers (Invitrogen) and Superscript II reverse transcriptase (Invitrogen) for cDNA synthesis, fragmentation and labelling. Hybridizations were performed at the Genome Québec Innovation Centre (McGill University, Montréal, Canada). Raw data were corrected for background signals using the RMA algorithm and quantile normalization^58^. Raw data were deposited to the Gene Expression Omnibus (GEO) public database (NCBI) under series entry “GSE117438”. Expression levels obtained from three replicates for each condition were compared using the FlexArray 1.3 software (Génome Québec, Mac Gill University). The 758 genes showing a P-value < 0.05 using the Empirical Bayes algorithm^59^, were further considered. Since the RMA algorithm decreases the false positive rate and compresses the fold change, a 2-fold change cut-off value was used for the determination of differentially expressed genes. Expression data for all differentially expressed genes is available in Supplementary Table S1. For microarrays validation, quantitative reverse transcription-PCR (qRT-PCR) experiments were conducted^57^ using primers described in Supplementary Table S4. PCR reactions were performed in triplicate and the standard deviations were lower than 0.15 CT.

### Proteomic analyses

Bacteria were grown in LB broth to mid-log phase (A_580_ = 0.4). Total proteins were extracted, quantified and digested as previously described^60^. All experiments were performed on a LTQ-Orbitrap Elite (Thermo Scientific) coupled to an Easy nLC II system (Thermo Scientific). One microliter of sample was injected onto an enrichment column (C18 PepMap100, Thermo Scientific). The separation was performed with an analytical column needle (NTCC-360/100-5-153, NikkyoTechnos, Japan). The mobile phase consisted of H2O/0.1% formic acid (FA) (buffer A) and CH3CN/FA 0.1% (buffer B). Tryptic peptides were eluted at a flow rate of 300 nL/min using a three-step linear gradient: from 2 to 40% B over 75 min, from 40 to 80% B in 4 in and 11 min at 80% B. The mass spectrometer was operated in positive ionization mode with capillary voltage and source temperature set at 1.5 kV and 275 °C, respectively. The samples were analyzed using CID (collision induced dissociation) method. The first scan (MS spectra) was recorded in the Orbitrap analyzer (R = 60,000) with the mass range m/z 400–1800. Then, the 20 most intense ions were selected for MS^2^ experiments. Singly charged species were excluded for MS^2^ experiments. Dynamic exclusion of already fragmented precursor ions was applied for 30 s, with a repeat count of 1, a repeat duration of 30 s and an exclusion mass width of ±10 ppm. Fragmentation occurred in the linear ion trap analyzer with collision energy of 35%. All measurements in the Orbitrap analyzer were performed with on-the-fly internal recalibration (lock mass) at m/z 445.12002 (polydimethylcyclosiloxane). After MS analysis, raw data were imported in Progenesis LC-MS software (Nonlinear Dynamics). For comparison, one sample was set as a reference and the retention times of all other samples within the experiment were aligned. After alignment and normalization, statistical analysis was performed for one-way analysis of variance (ANOVA) calculations. Peptide features presenting a *p*-value and a *q*-value less than 0.05, and a power greater than 0.8 were retained. MS/MS spectra from selected peptides were exported for peptide identification with Mascot (Matrix Science) against the database restricted to *P. aeruginosa* PA01 (http://www.pseudomonas.com). Database searches were performed with the following parameters: 1 missed trypsin cleavage site allowed; variable modifications: carbamidomethylation of cysteine and oxidation of methionine. Peptides with scores above 20 were imported into Progenesis. For each condition, the total cumulative abundance of the protein was calculated by summing the abundances of peptides. Proteins identified with less than 2 peptides were discarded. Only the proteins which varied by 2-fold in these average normalized abundances between growth conditions were retained. Expression data for all differentially expressed proteins is available in Supplementary Table S2.

### Functional profiling

The enrichment factor (EF) was calculated by comparing normalized PseudoCAP classes experimentally detected and normalized PseudoCAP classes annotated using the following formula: EF = (number of specific PseudoCAP classes detected/number of all PseudoCAP classes detected)/(number of specific PseudoCAP classes annotated/number of all PseudoCAP classes annotated). Functional categories displaying an *EF* ≥1.5 are defined as overrepresented in the functional transcriptomic or proteomic profiling of the *sigX* mutant.

### Anisotropy measurements

Fluorescence anisotropy analysis of *P. aeruginosa* cells grown to mid-log phase (A_580_ = 0.4) were performed as previously described^61^ with few modifications. Cell pellets were washed two times (7500 × g, 5 min, 25 °C) in 10 mM MgSO_4_ and resuspended in the same wash solution to reach an A_580_ of 0.1. One µL of a 4 mM of1,6-diphenyl-1,3,5-hexatriene (DPH) stock solution (Sigma-Aldrich) in tetrahydrofuran was added to 1 mL aliquot of the resuspended cultures and incubated in the dark for 30 min at 37 °C to allow the probe to incorporate into the cytoplasmic membrane. Measurement of the fluorescence polarization was performed using the Spark 20 M multimode Microplate Reader, equipped with an active temperature regulation system (Te-Cool^TM^, Tecan Group Ltd., Männedorf, Switzerland). Excitation and emission wavelengths were set to 365 and 425 nm, respectively, and the anisotropy *(r)* was calculated according to Lakowicz^62^. Three measurements were performed for each sample and data were recorded using SparkControl^TM^ software (Version 2.1, Tecan Group Ltd., Männedorf, Switzerland). The relationship between fluorescence polarization and membrane fluidity is an inverse one, where increasing anisotropy values correspond to a more rigid membrane and vice versa. All values are reported as means of triplicate analyses for each experimental variable.

### Lipids extraction and fatty acids methyl ester (FAME) analysis

Bacterial aliquots were collected in triplicate at the mid-log phase (A_580_ = 0.4) and pelleted by centrifugation. After removal of supernatant and three washes with sterile saline solution, bacteria were centrifuged at 4 °C (13,000 × g) for 15 minutes. Aliquots were resuspended in deionized water and lyophilized using a Heto PowerDry PL9000-50/HSC500 Freeze Dryer (Thermo Fisher Scientific, Saint-Herblain, France). Fatty acid methyl esters (FAME) were prepared by incubation for 15 min at 95 °C in a mix of 14% boron trifluoride (BF_3_)/methanol followed by extraction with hexane as described by Morrison and Smith^63^. FAMEs were separated and analyzed by gas chromatography (GC) coupled to flame ionization detection using an Agilent Technology, 6890 Network GC System, equipped with a split/splitless 7683 Series Injector. The samples were separated through a CP-Sil 88 capillary column (Chrompack, Middelburg, the Netherlands; length, 50 m; inner diameter, 0.25 mm; 0.25 mm film). FAMEs were identified by co-injection of reference standards obtained from Supelco (Bellefonte, Pennsylvania, USA) and were quantified on basis of their peak areas in total ion counts. The degree of FA saturation was determined as the ratio between the saturated FAs and the unsaturated FAs^64^. All experiments were performed in triplicate.

### Extraction and quantification of 2-alkyl-4-hydroxyquinolines (HAQs)

2-heptyl-4-hydroxyquinolone (HHQ) was extracted from cells grown to OD_580_ = 0.4 and quantified by liquid chromatography coupled to mass spectrometry (LC-MS/MS), as previously described^65^. Briefly, samples were analyzed by high-performance liquid chromatography (HPLC; Waters 2795, Mississauga, ON, Canada) equipped with a C8 reverse-phase column (Eclipse XDB-C8, Agilent Technologies, Mississauga, ON, Canada), and the detector was a mass spectrometer (Quattro Premier XE, Waters). The column was a 4.6 × 250 mm Agilent XDB-C8 column (particle size, 5 mm). The mobile phase was composed of water and acetonitrile containing 1% acetic acid. The initial solvent composition was 80% water: 20% acetonitrile, which was linearly changed to 2% water: 98% acetonitrile in 28 min, kept stable for an additional 6 min, returned to the initial values in 1 min, and finally left to stabilize for another 3 min. The flow rate was 0.4 ml/min, which was split to 40 µl through a Valco Tee splitter. The volume of the sample injected was 20 µl. The analyses were performed in positive electrospray ionization mode. The capillary voltage was 3 kV and the cone voltage, 21 V. The source temperature was 120 °C. Nitrogen was used as a nebulizing and drying gas at flow rates of 20 and 200 ml/min, respectively. Data were collected in Selective Ion Detection (SID) mode using the m/z 260 and 264 ions. Quantification of HHQ in bacterial cultures was performed by measuring the relative area of the chromatograms of the pseudomolecular ions of HHQ and of the internal standard (5,6,7,8-tetradeutero-4-hydroxy-2-heptylquinoline (HHQ-d4)) and multiplying the ratio by the concentration of the internal standard added.

### Statistical analysis

Statistical significance was evaluated using Prism GraphPad online tool (https://www.graphpad.com/quickcalcs/ttest1/). The data were statistically analyzed using two-sample unpaired *t*-test to calculate p values. The mean with SD or SEM were calculated and plotted.

### VennDiagrams generation

Venn diagrams and hypergeometric testing to assess the significance of the overlap were created using the *VennDiagram* R package (https://cran.r-project.org/package=VennDiagram)^66^. RNA-seq data of the *P. aeruginosa* PA14 *sigX* mutant and the PA14 overexpressing SigX^9^ were retrieved from the Gene Expression Omnibus (GEO) database using the accession number GSE50937. The transcriptomic profiling data of the *P. aeruginosa* PAO1 *sigX* mutant^8^ were retrieved from the Supplementary Table S1. The proteomic data of the *P. aeruginosa* PA14 strain overexpressing SigX^11^ were collected from Table 1 and Supplementary Table S2. The *P. aeruginosa* PAO1 orthologous genes/proteins of *P. aeruginosa* PA14 strain were identified using the reciprocal best Basic Local Alignment Search Tool hits implemented in the OrtholugeDB^67^. The transcriptomic and proteomic data obtained in this study and the all sets of collected data were used to draw the Venn diagrams displayed in the Supplementary Fig. S2.

## Electronic supplementary material

Supplementary Material

supplementary Table S1

supplementary Table S2`
