# Supplementary material for: The absence of SigX results in impaired carbon metabolism and membrane fluidity in Pseudomonas aeruginosa
Source: Sci Rep. 2018 Nov 21;8:17212. doi: 10.1038/s41598-018-35503-3 (PMC6249292; doi:10.1038/s41598-018-35503-3)
Supplement: Supplementary file 1 — Supplementary Material [file 41598_2018_35503_MOESM1_ESM.pdf]

Supplementary Material

The absence of SigX results in impaired carbon metabolism and membrane fluidity in *Pseudomonas aeruginosa*

Maud Fléchard[1°], Rachel Duchesne[1°], Ali Tahrioui[1], Emeline Bouffartigues[1] Ségolène Depayras[1], Julie Hardouin[2], Coralie Lagy[1], Olivier Maillot[1], Damien Tortuel[1], Cecil Onyedikachi Azuama[1], Thomas Clamens[1], Cécile Duclairoir-Poc[1], Manuella Catel-Ferreira[1], Gwendoline Gicquel[1], Marc G. J. Feuilloley[1], Olivier Lesouhaitier[1], Hermann J. Heipieper[3], Marie-Christine Groleau[4], Éric Déziel[4], Pierre Cornelis[1] and Sylvie Chevalier[1*]

Supplementary Figure S1. Correlation between the Log_2 fold change of several genes in RT-qPCR and microarray data.

Number (Graph)	PA number	Log_2 FC (DNA array)	Log_2 FC (RT-qPCR)
1	PA0427	-1,52	-1,77
2	PA0527	-2,12	-1,69
3	PA0779	1,39	1,81
4	PA0958	-1,14	-1,28
5	PA1053	-2,31	-2,95
6	PA1774	-2,45	-5,35
7	PA1775	-3,37	-2,69
8	PA3006	-4,55	-3,55
9	PA3038	14,88	7,88
10	PA3186	-2,81	-5
11	PA3405	1,05	1,48
12	PA4296	2,6	2,97
13	PA4306	1,06	1,96
14	PA4843	-2,98	-3,69
15	PA5091	3,02	1,85
16	PA5332	-2,15	-1,91

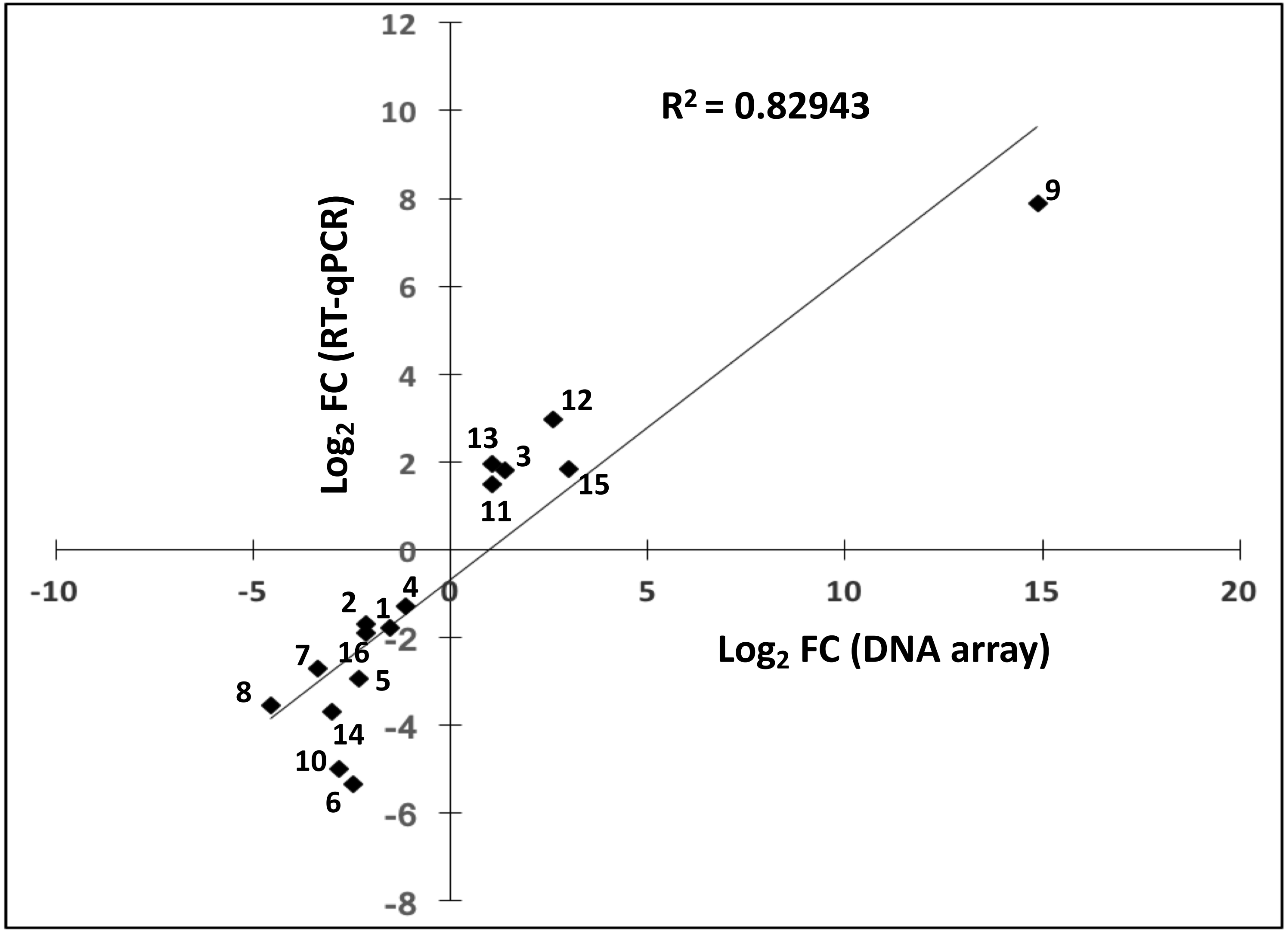

Supplementary Figure S2. Venn diagrams produced using the *VennDiagram* R package. (A) Venn diagram showing the overlap between transcriptomic and proteomic data of the *P. aeruginosa* PAO1 *sigX* mutant generated in this study. Hyper-geometrical testing was conducted to assess the significance of the overlap and the p-value is indicated (B) A four-set Venn diagram comparing transcriptomic data of PAO1 *sigX* mutant generated in this study (microarrays), PAO1 *sigX* mutant[8], PA14 *sigX* mutant[9], and PA14 overexpressing SigX[9] (C) A two-set Venn diagram displaying the overlap between PAO1 *sigX* mutant (this study, LTQ-orbitrap) and PA14 overexpressing SigX[11] proteomic data.

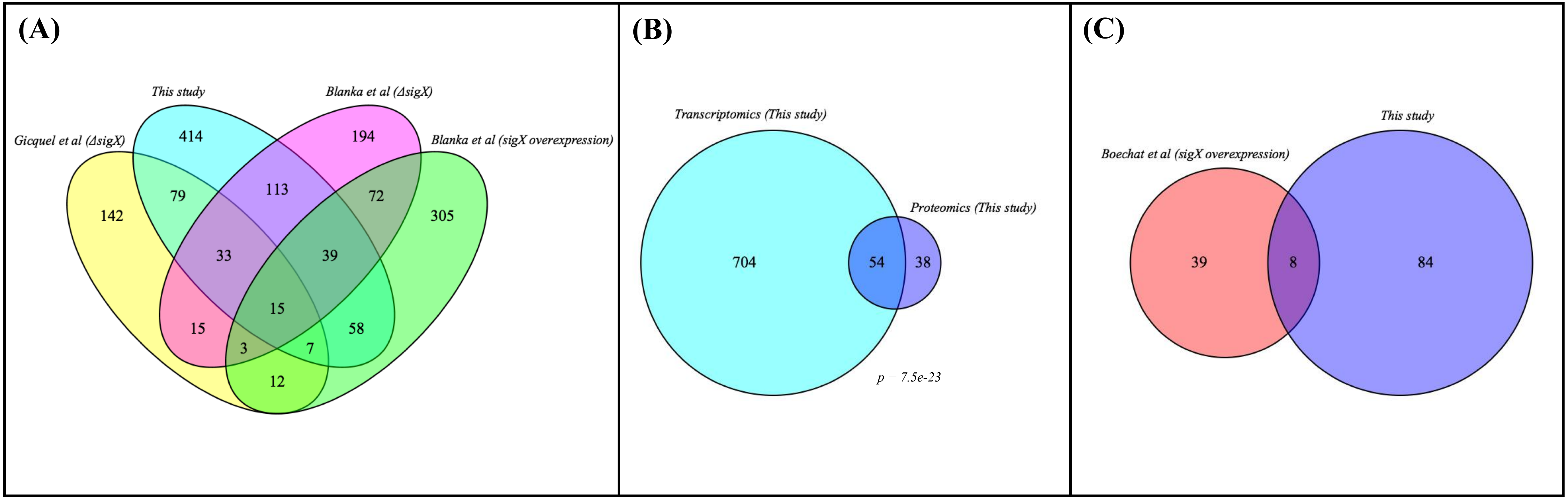

Supplementary Table S3. Selected up- and down-regulated genes (microarrays) and proteins (proteomics) in *P. aeruginosa* PAOSX (*sigX* mutant) versus H103 (WT). Only significantly dysregulated genes are included (p-value<0.05), and the fold change cut-off between PAOSX and H103 strains is 2. CbrAB-, Crc- and Hfq-regulated genes are indicated: CbrAB (*ΔcbrB*[36]); Crc (*Δcrc*[36]); Hfq[1] (*Δhfq*[40]), Hfq[2] (*Δhfq*[31]); Hfq[3] (Hfq targets, ChIP-seq[41]). Operons have been highlighted in grey. *: genes expression validated by qRT-PCR. NC: no expression change.

Locus	Gene name	Product name	PAOSX/H103		CbrA/B ; Crc ; Hfq
			mRNA	Protein	
**Transport of small molecules**					
Porins					
PA0162	*opdC*	Histidine porine OpdC	-7.7	-8.9	Hfq[1,2,3]
PA0291	*oprE*	Anaerobically-induced outer membrane porin OprE precursor	-2.2/-5*		Hfq[2], Crc
PA1178	*oprH*	PhoP/Q and low Mg^{2+} inducible outer membrane protein H1 precursor	-2		
PA2113	*opdO*	Pyroglutamate uptake porin	-5	2.9	Hfq[3]
PA3038	*opdQ*	Porin, OprD family	14.8/7.9*		
PA3186	*oprB*	Glucose/carbohydrate outer membrane porin OprB precursor	-2.7/-5*		Hfq[1,2] ; Crc
PA3790	*oprC*	Putative copper transport outer membrane porin OprC precursor	-4.35		Hfq[2]
PA4501	*opdD*	Glycine-glutamate dipeptide porin OpdD (OpdP)	3.2		Crc ; CbrA/B ; Hfq[1,2]
Carboxylic acid transporters					
PA0215	*madL*	Malonate transporter MadL	15.9		
PA0216	*madM*	Malonate transporter MadM	6.9		
PA0229	*pcaT*	Dicarboxylic acid transporter PcaT	7.6		Hfq[1]
PA1183	*dctA*	C4-dicarboxylate transport protein	-3.3		Crc
PA1863	*modA*	Molybdate-binding periplasmic protein precursor ModA	-3.45		
PA4616		Probable C4-dicarboxylate-binding protein (DctP family)	-2.44		
Carbohydrate transporters					
PA1946	*rbsB*	Binding protein component precursor of ABC ribose transporter	2.7		Crc, Hfq[1]

PA1947	*rbsA*	Ribose transport protein RbsA	4.7		Crc, Hfq[1]
PA1948	*rbsC*	Membrane protein component of ABC ribose transporter	3.3		Crc
PA2338	*mltE*	Probable binding protein component of ABC maltose/mannitol transporter	4.3		
PA2339	*mltF*	Probable binding-protein-dependent maltose/mannitol transport protein	3.9		
PA2340	*mltG*	Probable binding-protein-dependent maltose/mannitol transport protein	4.4		
PA2341	*mltK*	Probable ATP-binding component of ABC maltose/mannitol transporter	4		
PA3187	*gltK*	Probable permease of ABC sugar transporter	-10		Crc
PA3188	*gltG*	Probable permease of ABC sugar transporter	-7.7		Crc, Hfq[1]
PA3189	*gltF*	Probable permease of ABC sugar transporter	-2.9		Crc, Hfq[1]
PA3190	*gltB*	Probable binding protein component of ABC sugar transporter	-2		Crc
Amino acid transporters					
PA0295	/	Probable periplasmic polyamine binding protein	-3.33		
PA0603	*agtA*	ATP-binding cassette (ABC) transporter complex	8.7		
PA0604	*agtB*	ATP-binding cassette (ABC) transporter complex	14.8	3.8	Hfq[1,3]
PA0782	*putA*	Proline dehydrogenase PutA	-14.28		
PA0783	*putP*	Proline transporter	-4		Hfq[1,2]
PA0866	*aroP2*	Aromatic amino acid transport protein AroP2	3.95		CbrA/B ; Crc ; Hfq[1,2]
PA1074	*braC*	Branched-chain amino acid transport protein BraC	2.39		CbrA/B ; Crc ; Hfq[1,2]
PA1339	*aatP*	Amino acid ABC transporter ATP binding protein	2.6		Crc
PA1342	*aatJ*	ABC-type amino acid transport/signal transduction systems. Periplasmic component/domain	2.4		Crc ; CbrA/B, Hfq[1]
PA2711	*potF4*	Probable periplasmic spermidine/putrescine-binding protein	2.1		Hfq[1]
PA3641	/	Probable amino acid permease	-2.6		

PA3865	/	Probable amino acid binding protein	-2.43		Crc, Hfq[1]
PA3891	*opuC*	OpuC ABC transporter. ATP-binding protein	2.1		
PA4496	*dppA1*	Probable binding protein component of ABC transporter	5.29		CbrA/B ; Crc ; Hfq[1,2]
PA4497	*dppA2*	Probable binding protein component of ABC transporter	5.7		Hfq[1]
PA4910	/	Branched chain amino acid ABC transporter ATP binding protein	2.6		Hfq[1,2]
PA4913	/	Probable binding protein component of ABC transporter	5.1		Hfq[1,2] ; Crc
PA5094		Probable ATP-binding component of ABC transporter involved in histidine uptake	6.7		
PA5095		Probable permease of ABC transporter involved in histidine uptake	5.2		
PA5096		Probable ATP binding cassette involved in histidine uptake	3.8	18.8	Hfq[1]
PA5097		Probable amino acid permease involved in histidine uptake	5.2		Hfq[1]
PA5099	*hutI*	Probable transporter involved in histidine uptake	4.6		
PA5153		Lysine/arginine/ornithine/histidine/octopine) ABC transporter periplasmic binding protein	2.44		CbrA/B ; Hfq[1,2]
**Metabolisms**					
Fatty acid metabolism					
PA0492		Conserved hypothetical protein	-16.6		Crc
PA0493		Probable biotin-requiring enzyme	-11.1		
PA0494		Probable acyl-CoA carboxylase subunit	-6.25		
PA0495		Hypothetical protein	-8.3		
PA0496		Conserved hypothetical protein	-3.125		
PA1609	*fabB*	β-ketoacyl-ACP synthase I	-2.3		
PA1610	*fabA*	β-hydroxydecanoyl-ACP dehydratase	-3.7		
PA2966	*acpP*	Acyl carrier protein	-2.27		
PA2967	*fabG*	3-oxoacyl-[acyl-carrier-protein] reductase		-2.2	
PA2968	*fabD*	Malonyl-coA-[acyl-carrier-protein] transacylase		-2.3	

PA3639	*accA*	Acetyl-coenzyme A carboxylase alpha subunit	-2		Hfq[2]
PA3645	*fabZ*	(3R)-hydroxymyristoyl-[acyl carrier protein] dehydratase	-2.38		Hfq[2]
PA5174	*fabY*	β-ketoacyl synthase KASI/II domain condensation enzymes	-5.26	-8.9	
Amino acid metabolism					
PA0865	*hpd*	4-hydroxyphenylpyruvate dioxygenase /tyrosine assimilation	5.2		Hfq[2] ; Crc
PA0870	*phhC*	Aromatic amino acid aminotransferase	1.7	2.4	Crc ; Hfq[1,2,3]
PA0898	*aruD*	Succinylglutamate 5-semialdehyde dehydrogenase	2.3		CbrA/B ; Crc
PA0899	*aruB*	Succinylarginine dihydrolase	2.4		CbrA/B ; Crc
PA0901	*aruE*	Succinylglutamate desuccinylase	2.2		CbrA/B ; Crc
PA1337	*ansB*	Glutaminase-asparaginase	2.4	2.4	
PA1338	*ggt*	γ-glutamyltranspeptidase precursor	2.7		Hfq[1,2]
PA2011	*liuE*	3-hydroxy-3-methylglutaryl-CoA lyase	4.1		Crc
PA2012	*liuD*	Methylcrotonyl-CoA carboxylase, α-subunit	4		Crc, Hfq[1]
PA2013	*liuC*	Putative 3-methylglutaconyl-CoA hydratase	3.3		Crc, Hfq[1]
PA2014	*liuB*	Methylcrotonyl-CoA carboxylase, β-subunit	3.8		Crc, Hfq[1]
PA2015	*liuA*	Putative isovaleryl-CoA dehydrogenase	3.7		Crc, Hfq[1]
PA2247	*bkdA1*	2-oxoisovalerate dehydrogenase	4.4	2.7	CbrA/B ; Crc ; Hfq[1,2 ,3]
PA2248	*bkdA2*	2-oxoisovalerate dehydrogenase	5.6		CbrA/B ; Crc ; Hfq[1,2]
PA2249	*bkdB1*	Branched-chain alpha-keto acid dehydrogenase	6	2.2	CbrA/B ; Crc ; Hfq[1,2,3]
PA2250	*lpdV*	Lipoamide dehydrogenase-Val	6.4	2.9	CbrA/B; Crc; Hfq[1,2,3]
PA2862	*lipA*	Lactonizing lipase precursor	4.7		CbrA/B ; Crc ; Hfq[2]
PA3569	*mmsA*	Branched chain aminoacid assimilation	30		Hfq[1,2]
PA3570	*mmsB*	Branched chain aminoacid assimilation	33.1		Hfq[1,2]
PA4588	*gdhA*	Glutamate dehydrogenase	2.4		Hfq[1,2]
PA5091	*hutG*	*N*-formylglutamate amidohydrolase	3		CbrA/B ; Crc ; Hfq[2]

PA5092	*hutI*	Imidazolone-5-propionate hydrolase HutI	2.9		CbrA/B ; Crc ; Hfq[2]
PA5093		Probable histidine/phenylalanine ammonia-lyase	2.9		
PA5098	*hutH*	Histidine ammonia-lyase	32.7	15	CbrA/B ; Crc ; Hfq[1,2,3]
PA5100	*hutU*	Urocanase	14.4	5.9	CbrA/B ; Crc ; Hfq[2]
Carbon metabolism					
PA0153	*pcaH*	Protocatechuate 3.4-dioxygenase	4		Hfq[1,2]
PA0154	*pcaG*	Protocatechuate 3.4-dioxygenase	3.3		Hfq[1,2]
PA0208	*mdcA*	Malonate decarboxylase	11.1		
PA0209	*mdcB*	Conserved hypothetical protein	13.6		
PA0210	*mdcC*	Malonate decarboxylase	21.1		
PA0211	*mdcD*	Acetyl-CoA carboxylase	14.3	31.6	Hfq[3]
PA0212	*mdcE*	Malonate decarboxylase	5.2	44.4	Hfq[3]
PA0213	*mdcG*	Hypothetical protein	17.1		
PA0214	*mdcH*	Probable acyl transferase	12.4		
PA0226		Probable CoA transferase, subunit A	9.8		Hfq[1,2]
PA0227	*pcaJ*	Malonate decarboxylase	20.6	17.5	Hfq[1,2]
PA0228	*pcaF*	Probable CoA transferase.	13.9	16.1	Hfq[1,2]
PA0230	*pcaB*	3-carboxy-cis.cis-muconate cycloisomerase	4.2		
PA0231	*pcaD*	β-ketoadipate enol-lactone hydrolase	5.4		
PA0232	*pcaC*	γ-carboxymuconolactone decarboxylase	10.7		
PA0235	*pcaK*	4-hydroxybenzoate transporter PcaK	1.5		Hfq[1]
PA0609	*trpE*	Anthranilate synthetase, component I	1.7		
PA0887	*acsA*	Acetyl-coenzyme A synthetase	2.9		CbrA/B ; Crc
PA0996	*pqsA*	Probable coenzyme A ligase, PQS biosynthesis	-2.5		
PA0997	*pqsB*	PqsB, PQS biosynthesis	-2.7	-11.5	
PA0998	*pqsC*	PqsC, PQS biosynthesis	-2.38	-4.5	
PA0999	*pqsD*	3-oxoacyl-[acyl-carrier-protein] synthase III	-2	-6.1	
PA1001	*phnA*	Anthranilate synthase component I	-2		Crc
PA1002	*phnB*	Anthranilate synthase component II	-2		Crc

PA1892		Hypothetical protein	4.2		Hfq[1,2]
PA1893		Hypothetical protein	4		Hfq[1,2]
PA1894		Hypothetical protein	9.4		Hfq[1,2]
PA1895		Hypothetical protein	7.3		Hfq[1,2]
PA1896		Hypothetical protein	5.2		Hfq[1,2]
PA1897		Hypothetical protein	4.5		Hfq[1,2]
PA1901	*phzC2*	Phenazine biosynthesis protein PhzC	9.7	-30.4	Hfq[1]
PA1902	*phzD2*	Phenazine biosynthesis protein PhzD	10.15	-11.7	Hfq[1]
PA1903	*phzE2*	Phenazine biosynthesis protein PhzE	10.6	-16.2	Hfq[1]
PA1904	*phzF2*	Phenazine biosynthesis protein PhzF	11.6	-11.1	Hfq[1]
PA1905	*phzG2*	Probable pyridoxamine 5'-phosphate oxidase	11.8	-8.1	Hfq[1]
PA1978	*erbR*	Glycerol metabolism activator	2.4		Hfq[1,2]
PA1981		Hypothetical protein	4.3		Hfq[1,2]
PA1982	*exaA*	Quinoprotein ethanol dehydrogenase	4.5		Hfq[1,2]
PA1983	*exaB*	Cytochrome *c*550	7.5		Hfq[1,2]
PA1984	*exaC*	NAD+ dependent aldehyde dehydrogenase ExaC	2.4		Hfq[1,2] ; CbrA/B
PA2001	*atoB*	Acetyl-CoA acetyltransferase	3.9	3.3	Hfq[1,2,3]
PA2003	*bdhA*	3-hydroxybutyrate dehydrogenase	5.9	5.4	Hfq[1,2]
PA2507	*catA*	Catechol 1.2-dioxygenase	160	25.5	Hfq[1,2]
PA2508	*catC*	Muconolactone δ-isomerase	106		Hfq[1,2]
PA2509	*catB*	Muconate cycloisomerase I	7.3	8.5	Hfq[1,2]
PA2511	*antR*	Transcriptional regulator	10.1		Hfq[1,2]
PA2512	*antA*	Anthranilate dioxygenase	163.1	10.4	Hfq[1,2]
PA2513	*antB*	Anthranilate dioxygenase	502.1	13	Hfq[1,2]
PA2514	*antC*	Anthranilate dioxygenase reductase	140.4	5.4	Hfq[1,2]
PA2515	*xylL*	cis-1.2-dihydroxycyclohexa-3.4-diene carboxylate dehydrogenase	4.6		Hfq[1,2]
PA2516	*xylZ*	Toluate 1.2-dioxygenase electron transfer component	3.1		Hfq[1,2]
PA2517	*xylY*	Toluate 1.2-dioxygenase	5.5		Hfq[1,2]
PA2518	*xylX*	Toluate 1.2-dioxygenase	4.9		Hfq[1,2]

PA2519	*xylS*	Transcriptional regulator	3.1		Hfq[1,2]
PA3362	*amiS*	Hypothetical protein	2.1		Hfq[1,2]
PA3363	*amiR*	Aliphatic amidase regualtor	3.5		Hfq[1,2]
PA3364	*amiC*	Aliphatic amidase expression-regulating protein	3.4		Hfq[1,2]
PA3365		Probable chaperone	7.2		Hfq[1,2]
PA3366	*amiE*	Aliphatic amidase	8.4	2.6	Crc; Hfq[1,2,3]
PA3709		Probable major facilitator superfamily (MFS) transporter	2.3		Hfq[1,2]
PA4209	*phzM*	Phenazine specific methyltransferase	10.2		Hfq[1,2]
PA4496	*dppA1*	Binding protein component of ABC transporter	5.3		Hfq[1,2]
PA4661	*pagL*	Lipid A 3-O-deacylase	NC	3.3	Hfq[3]
PA4733	*acsB*	Acetyl-coenzyme B synthetase	2.7		CbrA/B ; Crc
PA4910		Putative ABC transporter protein	2.6		Hfq[1,2]
PA4913		Binding protein component of ABC transporter	5.1		Hfq[1,2] ; Crc
PA5056	*phaC1*	Poly(3-hydroxyalkanoic acid) synthase 1	2.8		Crc ; Hfq[2]
PA5057	*phaD*	Poly(3-hydroxyalkanoic acid) depolymerase	2.1		Crc ; Hfq[2]
PA5058	*phaC2*	Poly(3-hydroxyalkanoic acid) synthase 2	2.6		Crc ; Hfq[2]
PA5060	*phaF*	Polyhydroxyalkanoate synthesis protein PhaF	2.2		Crc ; Hfq[2]
PA5332	*crc*	Catabolite repression control protein	-2.2		Crc
PA5380	*gbdR*	Transcriptional regulator for glycine betaine catabolism	2.9		Hfq[1,2]
PA5435	*pycB*	Pyruvate carboxylase	-4.2	2.4	Hfq[3]
PA5543		Hypothetical protein	2.5		Hfq[2]
PA5545		Hypothetical protein	2.3		Hfq[2]
Energy metabolism					
PA0105	*coxB*	Cytochrome *c* oxidase.	13.2		Hfq[1]
PA0106	*coxA*	Cytochrome *c* oxidase.	15.7		Hfq[1]
PA0107	*coxI*	Conserved hypothetical protein	9.4		Hfq[1]
PA0108	*coIII*	Cytochrome *c* oxidase.	10.9		Hfq[1]
PA0792	*prpD*	Propionate catabolic protein PrpD	7,8		Crc
PA0793		Hypothetical protein	7,4		Crc

PA0794		Probable aconitate hydratase	4,8		Crc
PA0795	*prpC*	Citrate synthase 2	6,1		Crc
PA0796	*prpB*	Carboxyphosphonoenolpyruvate phosphonomutase	4,2		Crc
PA1172	*napC*	Cytochrome *c*-type protein NapC	4.8		
PA1173	*napB*	Cytochrome *c*-type protein NapB precursor	5.1		
PA1174	*napA*	Periplasmic nitrate reductase protein NapA	4.9		
PA1175	*napD*	NapD protein of periplasmic nitrate reductase	4		
PA1176	*napF*	Ferredoxin protein NapF	4.5		
PA1177	*napE*	Periplasmic nitrate reductase protein NapE	4.4		
PA1555	*ccoN*	Cytochrome *c* oxidase. Cbb3-type. CcoN	-5.26		
PA1556	*ccoO*	Cytochrome *c* oxidase. Cbb3-type. CcoO	-4.54		
PA1557	*ccoP*	Cytochrome *c* oxidase. Cbb3-type. CcoP	-2.63		
PA1561	*aer*	Aerotaxis receptor Aer	-4.37		
PA3872	*narI*	Respiratory nitrate reductase γ chain	-4.16		
PA3873	*narJ*	respiratory nitrate reductase δ chain	-7.14		
PA3874	*narH*	respiratory nitrate reductase β chain	-11.11		
PA3875	*narG*	respiratory nitrate reductase α chain	-33.33		Crc
PA3876	*narK2*	Nitrite extrusion protein 2	-20		
PA3877	*narK1*	Nitrite extrusion protein 1	-20		
PA3878	*narX*	Two-component sensor NarX	-1.47		
PA3879	*narL*	Two-component response regulator NarL	-2.56		

Supplementary Figure S3. The fatty acids anabolic pathways are altered in the sigX mutant strain. The genes that were under-expressed in the sigX mutant compared to the WT strain are highlighted in red.

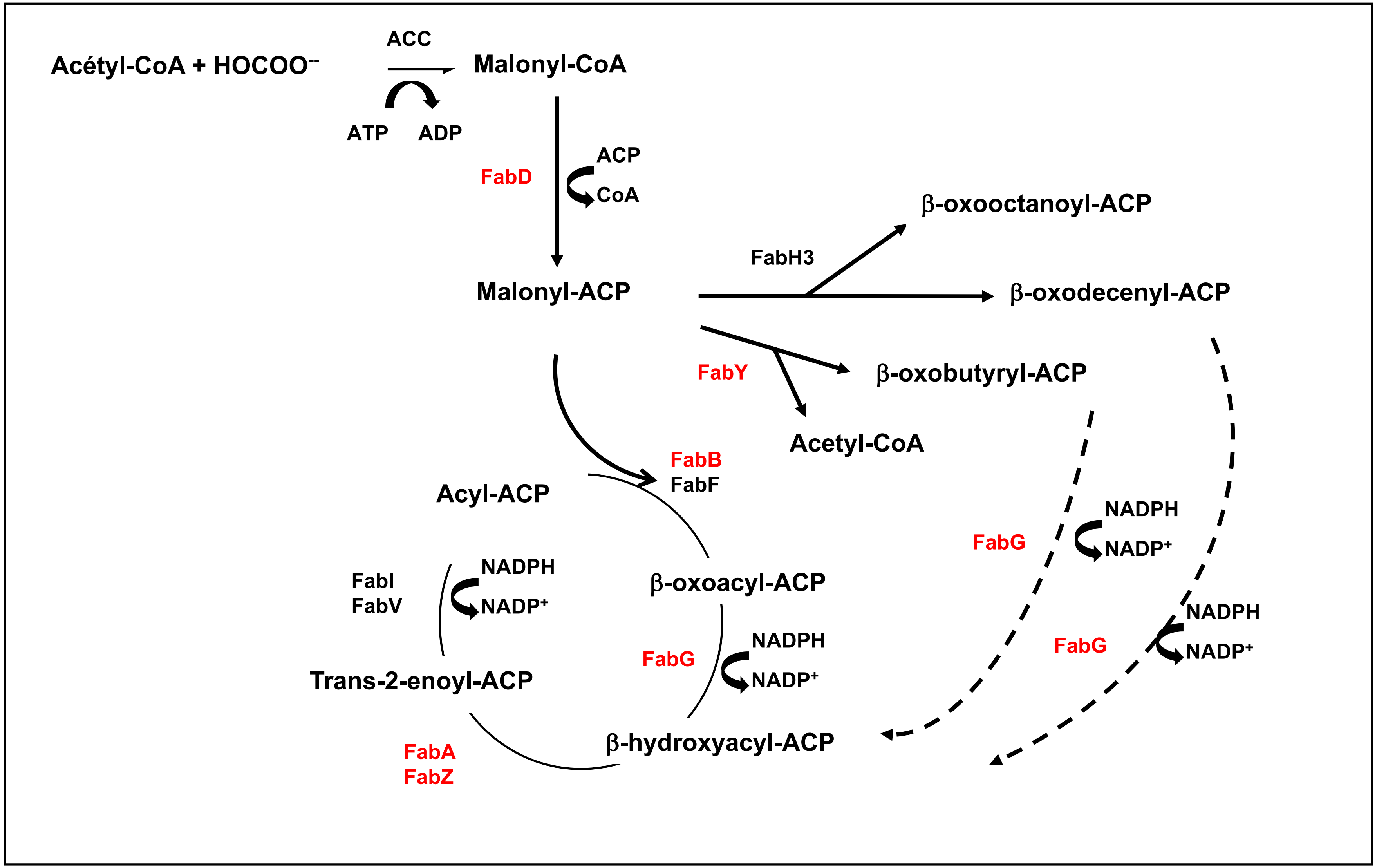

Supplementary Table S4: Primers used in this study

PA number	Gene name	Primer name	Sequence (5’-3’)
PA0291	*oprE*	FoprE	GGCGATGGCGAAGGTTCTA
		RoprE	CGCTGGAACTTGCCGATCT
PA0527	*dnr*	Fdnr	TTCCGGCTGCGTGAAGAT
		Rdnr	GCCTCGGCGAAGGTGTT
PA0779	*asrA*	FasrA	CCGCTTCAGTCCCAACGA
		RasrA	GCGGCAAGACCTTCTCCAT
PA0894	*aruB*	FaruB	GCGAGGTGCTGTTCCATCA
		RaruB	CAGTTCGGCAAGGACCTTCTC
PA0958	*oprD*	FoprD	CTACGGCTACGGCGAGGAT
		RoprD	CACGTACTTGGCTTCGAGGTT
PA1053		FPA1053	GGCGGCTGCCAATCC
		RPA1053	GGGCTTCCTCACGGCTGTA
PA1774	*cfrX*	FcfrX	CTGCGGGACCTGCTCAAG
		RcfrX	GCCGACCTGGCGATTG
PA1775	*cmpX*	FcmpX	TCGCCGGGCAGATCATT
		RcmpX	CACCTGTTGTCCGACCTGATAC
PA1777	*oprF*	FoprF	GCGTACAGCTGGACATGAAG
		RoprF	TTCATGAAGTCAGCCAGGTTCTT
PA2113	*opdO*	FopdO	CGGCTACCAGCGGATCAGT
		RopdO	GTAGCGCAGTTGCCAGGAA
PA2513	*antB*	FantB	GCGTCGAACAGTTCCTCTACC
		RantB	CTGCTCGGACTCCCACTG
PA3006	*psrA*	FpsrA	CGATTTCGGCGTGAACACTT
		RpsrA	CGAAGAACGGCACCATCAG
PA3038	*opdQ*	FopdQ	CGCCAACAAGGACGAGAAAT
		RopdQ	GGTCAGCAGGTCGATGTTGTC
PA3186	*oprB*	FoprB	GGACGCTACACCGACCAGTT
		RoprB	ATGCGGTCGTTGGAAAGGT
PA3366	*amiE*	FamiE	GGCGAGGAGGAAATGGGTAT
		RamiE	GTGGTTCTGCGACTGATCGTT
PA3405	*hasE*	FhasE	TGGACGAGAAGCGTTTTTCC
		RhasE	GAGACCCCGACACCCTTGT
PA4296	*pprB*	FpprB	TCGTCAAGGTCCTGGTTTCG
		RpprB	GGCGATGGTGGTGAGGAA
PA4306	*flp*	Fflp	GCAAACGCCATCGAATACG
		Rflp	TGGGACTCAATACGGCAATCA
PA4525	*pilA*	FpilA	TTGCCATTCCCCAGTATCAGA
		RpilA	CAGCGCCGAAGCACCTT
PA4726.11	*crcZ*	FcrcZ	AGCGCACAGACTGGTTGGAT
		RcrcZ	GCTGGGAGTTCAATAGCAAACG
PA4843	*gcbA*	FPA4843	CGGCGGACAGGTAGATGATC
		RPA4843	CCTGGGCACCGAATTGG
PA5091	*hutG*	FhutG	ACCGATACCGACTGGCACAT
		RhutG	TCGGAAGGGCGGTTGAG
PA5332	*crc*	Fcrc	TGATCAGCGGCTTAGGTTTC
		Rcrc	TTCTGGTTCAAGCTCTCGTC
16SRNA		F16S	AACCTGGGAACTGCATCCAA
		R16S	CTTCGCCACTGGTGTTCCTT
